# HArmonized single-cell RNA-seq Cell type Assisted Deconvolution (HASCAD)

**DOI:** 10.1186/s12920-023-01674-w

**Published:** 2023-10-31

**Authors:** Yen-Jung Chiu, Chung-En Ni, Yen-Hua Huang

**Affiliations:** 1 Institute of Biomedical Informatics, National Yang Ming Chiao Tung University, Taipei, 112 Taiwan; 2 Department of Biomedical Engineering, Ming Chuan University, Taoyuan, 333 Taiwan; 3 Center for Systems and Synthetic Biology, National Yang Ming Chiao Tung University, Taipei, 112 Taiwan

**Keywords:** Harmonization, Cell composition deconvolution, RNA-seq, Deep learning

## Abstract

**Background:**

Cell composition deconvolution (CCD) is a type of bioinformatic task to estimate the cell fractions from bulk gene expression profiles, such as RNA-seq. Many CCD models were developed to perform linear regression analysis using reference gene expression signatures of distinct cell types. Reference gene expression signatures could be generated from cell-specific gene expression profiles, such as scRNA-seq. However, the batch effects and dropout events frequently observed across scRNA-seq datasets have limited the performances of CCD methods.

**Methods:**

We developed a deep neural network (DNN) model, HASCAD, to predict the cell fractions of up to 15 immune cell types. HASCAD was trained using the bulk RNA-seq simulated from three scRNA-seq datasets that have been normalized by using a Harmony-Symphony based strategy. Mean square error and Pearson correlation coefficient were used to compare the performance of HASCAD with those of other widely used CCD methods. Two types of datasets, including a set of simulated bulk RNA-seq, and three human PBMC RNA-seq datasets, were arranged to conduct the benchmarks.

**Results:**

HASCAD is useful for the investigation of the impacts of immune cell heterogeneity on the therapeutic effects of immune checkpoint inhibitors, since the target cell types include the ones known to play a role in anti-tumor immunity, such as three subtypes of CD8 T cells and three subtypes of CD4 T cells. We found that the removal of batch effects in the reference scRNA-seq datasets could benefit the task of CCD. Our benchmarks showed that HASCAD is more suitable for analyzing bulk RNA-seq data, compared with the two widely used CCD methods, CIBERSORTx and quanTIseq. We applied HASCAD to analyze the liver cancer samples of TCGA-LIHC, and found that there were significant associations of the predicted abundance of Treg and effector CD8 T cell with patients’ overall survival.

**Conclusion:**

HASCAD could predict the cell composition of the PBMC bulk RNA-seq and classify the cell type from pure bulk RNA-seq. The model of HASCAD is available at https://github.com/holiday01/HASCAD.

**Supplementary Information:**

The online version contains supplementary material available at 10.1186/s12920-023-01674-w.

## Introduction

RNA sequencing (RNA-seq) is a next-generation sequencing-based technology to target whole transcriptome of a sample [1]. This technology has been widely used in large-scale disease studies, since it enables a sensitive detection of the global gene expression signatures of samples. However, the samples used for the RNA-seq analysis are usually prepared in the form of bulk tissues which might consist of various types of cells. Such a bulk-tissue RNA-seq approach measures only the average gene expression profiles (GEP) of various cell types contained in a tissue.

Notably, certain types of immune cells infiltrating in the tumor microenvironment (TME) may actively interact with cancer cells and thus promote malignant phenotypes such as enhancing survival of cancer cells and supporting their metastasis [2, 3]. The abundance and density of these tumor-infiltrating immune cells (TIICs) have been implicated in patient survival and anti-cancer treatment efficacy [4]. For example, macrophage cells are able to be polarized into classically activated macrophages (M1), or alternatively, they are able to become activated macrophages (M2). M2 cells promote the growth of cancer cells, while M1 cells inhibit the differentiation of cancer cells [5]. A few studies reported that a high M1/M2 ratio is associated with better survival of cancer patients [6, 7]. In addition, CD8 T cell and its functional subsets have been highlighted in many studies due to their roles in cancer immunotherapy [8]. A higher density and a greater abundance of CD8 T cells in the tumor microenvironment (TME) have been associated with a better prognosis for cancer patients [9]. In addition, Tu et al*.* analyzed the ICOS^+^ FOXP3^+^ regulatory T cells (Tregs) in 57 HCC patients by immune-histochemistry (IHC), and reported that the infiltration of ICOS^+^ FOXP3^+^ Tregs showed a negative correlation with patient survival [3].

Thus, to further investigate the biological roles played by various immune cells in complex tissues, a few computational methods have been developed to perform cell composition deconvolution (CCD) [10–15]. CCD methods that apply regression-based deconvolution need a set of pre-compiled reference GEP (refGEP) composed of cell-specific gene expression signatures. The refGEP could be derived from whole transcriptome datasets, including microarray, RNA-seq, and single-cell RNA-seq (scRNA-seq). Compared to other sources of reference data, scRNA-seq is likely to provide the specific gene expression signature of more diverse cell types, and thus scRNA-seq has become one of the favorite choices for buiding refGEP. Several CCD methods have reported high deconvolution accuracy by using scRNA-seq derived refGEP [13–16]. However, there are usually significant batch effects across scRNA-seq datasets derived from different studies, and thus directly pooling multiple scRNA-seq datasets to build refGEP may result in poor performance in CCD tasks.

To mitigate the influence caused by the technical biases in the data, quite a few methods, such as DWLS, CIBERSORTx, and MuSic, have chosen to build their refGEP with only a single scRNA-seq dataset, although this approach might limit the performance of CCD. Evidence suggests that leveraging heterogeneity across multiple reference datasets may reduce technical and biological biases in the data and thus increase the accuracy of CCD [17]. Therefore, SCDC proposed an ENSEMBLE deconvolution approach to integrate CCD results from different scRNA-seq datasets, which might implicitly reduce the confounding caused by cross-sample batch effects.

On the other hand, deep neural network (DNN)-based approaches, such as Scaden [12], have recently been applied to perform the task of CCD. It was asserted that hidden layers of DNN might represent a high-order encoding of the gene expression signatures of distinct cell types, which might be more robust to input noise and technical bias. The work of Scaden suggests that a deep learning-based CCD method could be trained with a huge set of simulated bulk RNA-seq data, which is prepared by subsampling and subsequently merging of cells obtained from single-cell RNA-seq datasets [12]. Nonetheless, in terms of analyzing complex tissues, the application of Scaden is restricted since it could predict the fractions of no more than 10 cell types [12]. Therefore, this study aims to create a DNN-based CCD method that is able to predict the fractions of more than 10 types of immune cells in the bulk RNA-seq samples.

In addition, we argue that the preparation of the huge training set should take the removal of batch effects across different reference datasets into consideration. Above all, training data should be derived from multiple sources and each may contain within-sample technical and biological biases. Although SCDC appears to address the issue of batch effects by using an ENSEMBLE approach, their results imply that the best performance of SCDC can be achieved only when the reference data and the bulk samples were derived from the same source. The ENSEMBLE approach adopted by SCDC did not really remove batch effects in the reference dataset.

Batch effects pose a great challenge to the analysis of high-throughput datasets, since such technical variations may confound the biological variations of interest in the down-stream statistical analysis. Tran et al*.* assessed 14 batch-effect correction methods for scRNA-seq data, and they recommend that “Harmony” is the first choice to try [18]. Harmony is designed to integrate scRNA-seq datasets derived from different technologies and multiple species [19]. Harmony projects the gene expression into a low-dimension embedding, and then iteratively adjusts the embedding in order to remove batch-specific variations, and thus enables the clustering to better correspond to distinct cell types. Hence, we used Harmony to preprocess the scRNA-seq datasets that were used to create the training set for our DNN model, HASCAD.

Hence in this study, Harmony, an algorithm that has been designed to normalize the scRNA-seq data, was used to remove the batch bias in the preparation of the training set [19]. This study builds a DNN-based CCD model that is trained by using the simulated gene signature matrix derived from the Harmony-normalized scRNA-seq data.

## Materials and methods

### The workflow

This study used the harmonized expression data of scRAN-seq to develop a CCD model, HASCAD (HArmonized ScRNA-seq Cell Assisted cell Deconvolution model). Our model is a deep neural network (DNN) model, which is inspired by the work of Scaden. Notably, our CCD model is designed to predict from the bulk RNA-seq data the fractions of 15 cell types (Table 1), including activated dendritic cell (aDC), plasmacytoid dendritic cell (pDC), memory and naïve B cells (bmem and bnaive), memory and naïve CD4 T cells (cd4mem and cd4naive), regulatory T cells (treg), effector, memory, and naïve CD8 T cells (cd8eff, cd8mem and cd8naive), hematopoietic stem cell (HSC), megakaryocyte (MK), CD14 monocyte (mono14), CD16 monocyte (mono16), and nature killer cells (NK).

**Table 1:** The size of samples of each immune cell type for three scRNA-seq datasets

	PBMC6K (3’ v1)	PBMC8K (3’ v2)	PBMC5GEX (5’)
*aDC*	76	210	124
*Memory B*	197	450	429
*Naïve B*	386	774	747
*Memory CD4 T*	600	818	868
*Naïve CD4 T*	983	1436	1109
*Effector CD8 T*	553	922	551
*Memory CD8 T*	55	161	240
*Naïve CD8 T*	299	899	254
*HSC*	21	20	23
*MK*	22	49	49
*CD14 monocyte*	857	1824	2248
*CD16 monocyte*	332	225	330
*NK*	296	323	304
*pDC*	11	68	74
*Treg*	82	97	119

**Fig. 1:**
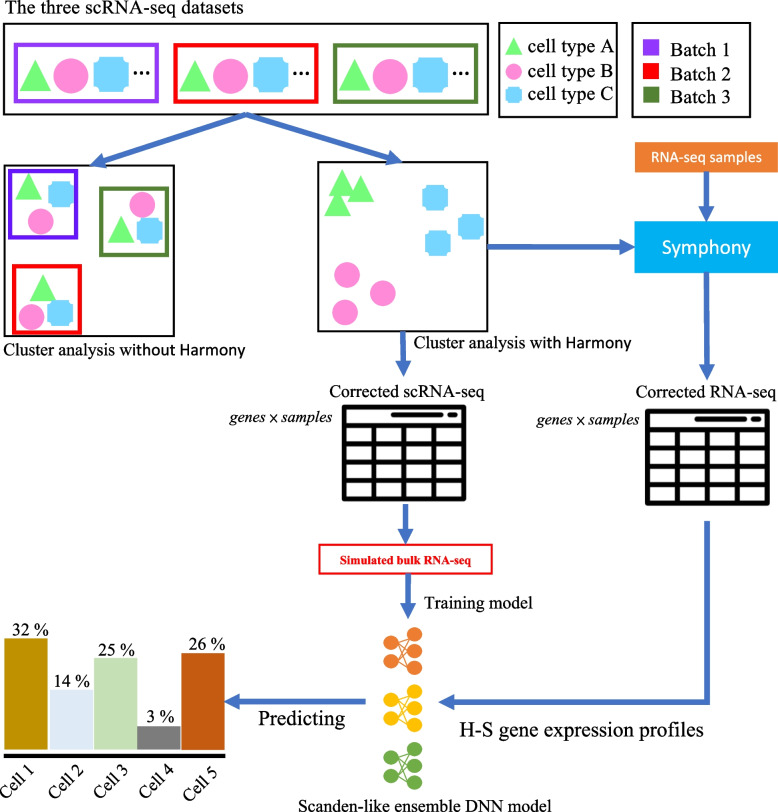
The workflow of HASCAD

The workflow of the build of our model is illustrated in Fig. 1. To start with, in order to prepare a huge dataset to train our DNN model, public scRNA-seq datasets of peripheral blood mononuclear cell (PBMC) that could provide the cell type-specific GEPs of the aforementioned 15 cell types were obtained. These datasets were used to simulate bulk RNA-seq samples, with each consisting of known fractions of various cell types.

Next, to integrate the GEPs derived from different scRNA-seq datasets, the expression values were preprocessed in order to remove batch-specific systematic variations. We chose to use Harmony, which is an algorithm designed to perform cross-sample normalization and batch effect removal in scRNA-seq datasets [19].

We carried out the procedures of Harmony to perform a PCA-based dimension reduction of the input data, and then the low-dimension embedding was iteratively adjusted [19]. The iteration was converged when different cell types were grouped separately, even though cells of the same type were derived from different batches (Fig. 1, the output of Harmony). Then the low-dimension embeddings in which batch variations have been cleaned were transformed back to cell-specific GEPs (Fig. 1, the matrix of *genes* $\times$ *samples* output by Harmony). These GEPs of single cells were used to simulate the training data, with the cell fractions being randomly assigned.

Finally, in terms of the cell fraction prediction of new bulk RNA-seq data, the expression values will be preprocessed by using the functions provided by Symphony, which is an extension tool of Harmony that is designed to integrate new datasets into reference atlases [20]. Hence, HASCAD predicts the cell composition in the new bulk RNA-seq data whose batch effects are removed by using Symphony (Fig. 1, the matrix of *genes* $\times$ *samples* output by Symphony).

### Preprocessing and normalization of scRNA-seq datasets

In order to prepare the training data, three PBMC scRNA-seq datasets, PBMC6K (3′ v1), PBMC8K (3′ v2), and PBMC5GEX (5′), were obtained from the GitHub repository of Symphony. These data were initially transformed by log_2_(CP10K + 1) using R package Seurat [19, 20]. CP10K is the count per million (CPM), whereas log_2_(CP10K + 1) is a standard transformation used in scRNA-seq data analysis [19]. The datasets PBMC8K and PBMC6K were generated by using Chromium Single Cell 3′ v2 chemistry and Chromium Single Cell 3′ v1 chemistry, respectively [20]. Chromium Single Cell 5′ paired-end chemistry was used to generate PBMC5GEX [18]. As these three datasets were generated by different assay chemistry, correction of the batch effects is necessary.

Before carrying out the Harmony-based normalization, variance stabilizing transform (VST) was used to select the genes and scale the gene expression for a dataset, which is the default setting of Harmony. Then, the functions implemented in Harmony were used to remove the batch-specific variations from the embedding, $\Sigma {V}^{T}$ [19, 20]. *U* is the PCA loading, the coefficients/weights of the liner combinations. For batch variation removal, the corrected embedding *Z'* would be calculated by iterating between the step of a maximum diversity clustering and the step of a mixture model based linear batch correction. The convolution of *U* with *Z’* was taken as the corrected gene expression profiles of scRNA-seq, which can then be used for the downstream cross-sample analyses.

$$M=U\Sigma {V}^{T}$$

$$Z=\Sigma {V}^{T}$$

To allow an integrated analysis of new scRNA-seq datasets with the batch effect-removed reference scRNA-seq, we used the query-projection strategy that was provided by Symphony [20]. A non-reference scRNA-seq set obtained from other studies was referred to as a query, and the functions of Symphony [20] could project the query data to the PC (principal component) space of the reference scRNA-seq data. The query embeddings (${Z}_{q}$) was calculated, and thus the query gene expression data could be projected as ${UZ}_{q}$. Identically, Symphony output $U{Z}_{q}{\prime}$, which was the corrected query gene expression profiles of RNA-seq. Hence, the projected gene expression profiles of the query scRNA-seq data could be integratedly analyzed with the gene expression profiles of the reference scRNA-seq data, and thus we used the harmonized datasets in the training and validation of the DNN of HASCAD (Table 2).

**Table 2:** Pre-processing of real bulk RNA-seq datasets as the input for HASCAD

	HASCAD
No H–S correction	Log_2_-transformed TPM
H–S correction	TPM corrected by the H–S approach

To assess the benefit of using Harmony-Symphony batch-corrected data (H–S corrected data) in the training of our CCD model, we also prepared non-harmonized scRNA-seq data. Non-harmonized data were pre-processed using a log transformation of the total count per-million (TPM) as follows:

$${Log}_{2}(TPM\;gene\;expression+1)$$

On the other hand, we also assessed if the Harmony-Symphony batch-corrected data could somehow be analyzed by other CCD methods, in a way to improve the performances. Since input data required for running CIBERSORTx and quanTIseq must be TPM values, we prepared the H–S corrected scRNA-seq data and transformed them back to TPM-like values (Table 3).

**Table 3:** Pre-processing of simulated bulk RNA-seq data as the input for CIBERSORTx and quanTIseq

	CIBERSORTx	quanTIseq
No H–S correction	TPM	TPM
H–S correction	TPM corrected by the H–S approach, and then transformed again back to TPM-like values	

### The architecture of our deep learning model

**Fig. 2:**
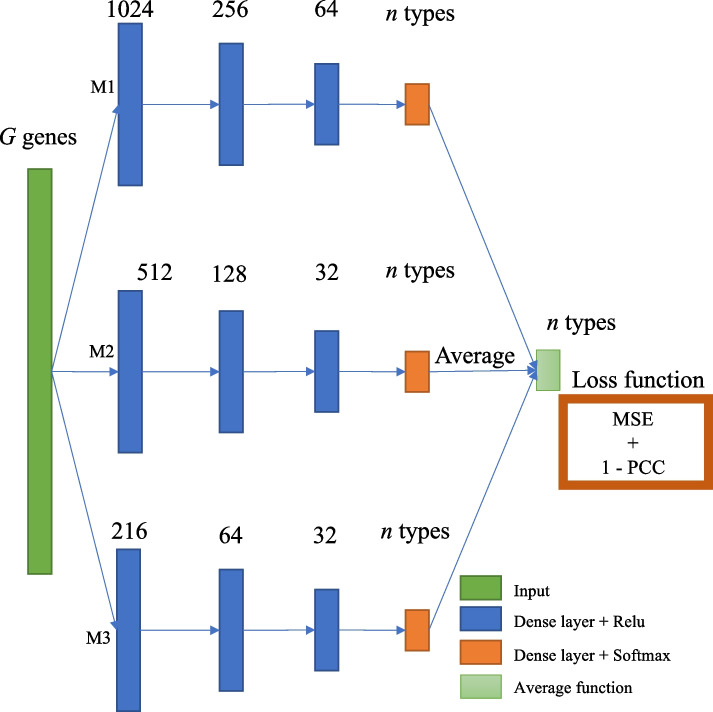
Architecture of the HASCAD model

The architecture of HASCAD is an ensemble of three parallel DNN modules (Fig. 2), which is similar to that used by Scaden [12]. Each module consists of three hidden layers in which the number of nodes is assigned between 32 and 1024. The node numbers for the first layers are 1024, 512, and 256, respectively. In the output layer, a softmax function is used to turn the sum of the predicted cell fractions to 1, making the outputs as non-negative values. The predicted cell fractions for each sample are the averaged values of the outputs from the three DNN modules. The optimization method is Adam, with a learning rate 0.0001 and a batch size 64. Unlike Scaden, we chose to use the loss functions composed of mean square error (MSE) and Pearson’s correlation coefficient (PCC). The model training opted early stop after 20 epochs by evaluating validation data.

The loss function used in the training of HASCAD was defined as follows:

$$argmin\;HASCAD\;loss\;function={MSE\;loss}+{PCC\;loss}$$

$$MSE\;loss=\frac{\sum\nolimits_1^s\left({truth}_s-{predict}_s\right)^2}{s}$$

$$PCC\;loss=1-r\left({truth},{predict}\right)$$

where *s* refers to each cell type; *truth*_*s*_ refers to the ground-truth cell proportion of cell *s*; $r$ in *PCC loss* refers to Pearson’s correlation coefficient.

### The preparation of the simulated bulk RNA-seq data for model training

We prepared the bulk RNA-seq data by using the Harmony-Symphony corrected scRNA-seq profiles. By following the strategy that has been used by Scaden, we simulated bulk RNA-seq data by combining the genes expression values from multiple single cells. In the simulated bulk RNA-seq data, *S*_*n*_ is a vector of *n*_*1*_, *n*_*2*_, …, where each element corresponds to the cell fraction assigned to each cell type. The cell fractions for various cell types in the samples are randomly chosen, and the gene expression values of the simulated bulk RNA-seq data correspond to the mean values of gene expression obtained from these samples. The ground truth *S*_*n*_ was normalized to sum to one:

$${{\varvec{S}}}_{{\varvec{n}}}=[n^1,n^2,\dots ]$$

$$\mathrm{Ground}\;\mathrm{truth}\;=\frac{{\mathbf S}_{\mathbf n}}{\mathbf\Sigma{\mathbf S}_{\mathbf n}}$$

### Benchmarks to assess the performance of HASCAD and other CCD methods

In the first benchmark we used the simulated gene expression profiles based on scRNA-seq data. ScRNA-seq gene expression profiles were used to generate in silico bulk RNA-seq data. To assess if Harmony-Symphony correction (H–S correction) could improve the performance of CCD, the model predicted the cell fractions in H–S corrected gene expression profiles and in non-H–S corrected gene expression profiles, respectively. Two scRNA-seq datasets were used to generate the training and validation data, and another dataset was used to make testing data.

The second benchmark used a set of bulk RNA-seq data of pure type immune cells retrieved from NCBI GEO (accession number GSE141498) [21]. There are the four types of immune cells in this dataset, including B cells, CD4 T cells, dendritic cells, and monocytes. The gene expression values in this dataset are provided as TPM counts. This assessment is to evaluate if HASCAD could be used to predict the cell compositions of the bulk RNA-seq sets consisting of pure-type immune cells. As a comparison, CIBERSORTx and quanTISeq were assessed by using the same datasets.

Next, HASCAD was assessed by using human PBMC RNA-seq dataset with experimentally estimated cell fractions. The dataset was GSE107572, downloaded from NCBI GEO [11]. The raw read counts of the RNA-seq expression were transformed by transcripts-per-millions (TPM) normalization:

$$\mathrm{TPM }=\frac{exp}{\sum exp}\times {10}^{6},$$

where *exp* denotes raw count reads mapped to transcript.

To apply HASCAD to predict cell fractions, the gene expression values for input were further transformed by using Harmony-Symphony correction. The performance metrics is the mean squared error (MSE) between the ground-truths and the predicted cell fractions. Two widely used CCD methods, CIBERSORTx and quanTIseq, were also assessed by using the original TPM values provided in the dataset, as suggested by the manuals of these two methods.

### Preprocessing of the bulk gene expression profiles of TCGA-LIHC samples

To predict the cell composition of HCC samples and to perform survival analysis of the patients, 414 RNA-seq samples of the Cancer Genome Atlas Liver Hepatocellular Carcinoma (TCGA-LIHC) dataset [22] were downloaded from NCI Genomic Data Commons (NCI GDC, https://gdc.cancer.gov/). R package biomaRt was used to map the Ensembl transcript ID to HGNC gene symbols, and the HTseq-count values were transformed using TPM normalization. By following the H–S correction procedure as described in Sect. 2.2, the TCGA-LIHC bulk RNA-seq data were deconvolved using HASCAD by adopting the three PBMC scRNA-seq datasets as the reference data. Kaplan–Meier plotter was utilized to investigate the association between immune cell abundance and the prognosis of LIHC patients. The data used in the survival analysis included the overall survival time of patients, and the predicted high/low abundance of various immune cell types.

## Results

### Training the HASCAD model

#### Preparation of the training data

**Fig. 3:**
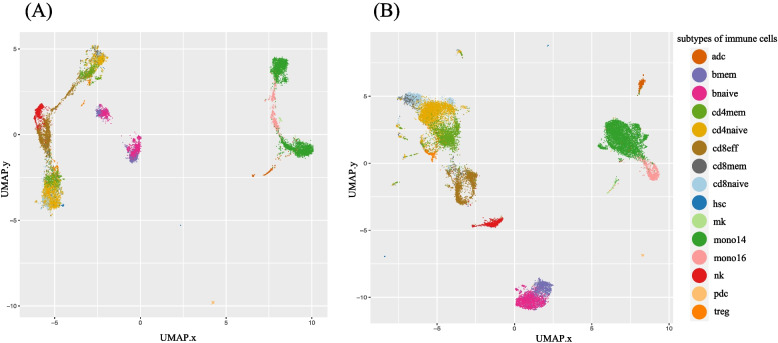
UMAP plots of (**A**) non-harmonized scRNA-seq data, (**B**) harmonized scRNA-seq data

Three scRNA-seq datasets, PBMC6K PBMC8K, and PBMC5GEX, were used to prepare the training data, and the numbers of cells for each dataset is provided in Table 1. By using the variance stabilizing transform (VST) method, a set of 2371 non-redundant genes with the variability more stable across a wide range of expression values were selected for the model training. The expression values of these genes were then normalized by using the functions of Harmony in order to remove batch-specific variations. To assess the impact of the normalization, UMAP plots were generated to contrast the clustering of cells across the three scRNA-seq datasets for model training, by using the non-corrected expression values and by using the corrected expression values (Fig. 3). The plot using the scRNA-seq data without pre-processing using the Harmony-Symphony functions is referred to as *non-harmonized* (Fig. 3A), whereas the plot of the scRNA-seq data pre-processed using the Harmony-Symphony approach is referred to as *Harmonized* (Fig. 3B). It can be seen that the normalization by Harmony is able to remove the batch variations and thus makes the clustering of cells more coherent to cell types. When the scRNA-seq data have not been normalized, the same types of cells were dispersed in different clusters (Fig. 3A), where the cluster separation appears to reveal the distinct batches of the cells. Conversely, after the scRNA-seq data being normalized by Harmony, cells derived from different batches were clustered largely consistent with their respective cell types (Fig. 3B). For example, memory and naïve B cells were clustered; mono14 cells (CD14^+^) and mono16 (CD16^+^) cells were clustered. Naïve CD8 T cells and memory CD8 T cells were clustered. Hence, we show that the Harmony-Symphony approach used in this study could remove to a certain extent the batch variations across scRNA-seq datasets, and thus we took the H–S corrected scRNA-seq data to prepare for the training and validation of our HASCAD model.

### Training of HASCAD model and the initial assessment

Thus, with the pre-processed scRNA-seq data, it was ready to train our HASCAD. To assess if the normalization of scRNA-seq data by using the Harmony-Symphony strategy can truly benefit the training of our HASCAD model, this study compared the performance metrics of the model trained by using H–S corrected scRNA-seq data, with that of the model that was trained by using the non-corrected scRNA-seq data. In the following text, the previous type of models will be referred to as Harmony-corrected model, and the latter ones will be referred to as non-Harmony-corrected models. A leave-one-dataset-out cross-validation was carried out as described in [12]. The Harmony-corrected models were trained by using the simulated bulk RNA-seq data that were generated based on either two of the three PBMC scRNA-seq datasets, and the models were evaluated by using the simulated bulk RNA-seq data that were generated from the remaining one PBMC scRNA-seq dataset.

**Fig. 4:**
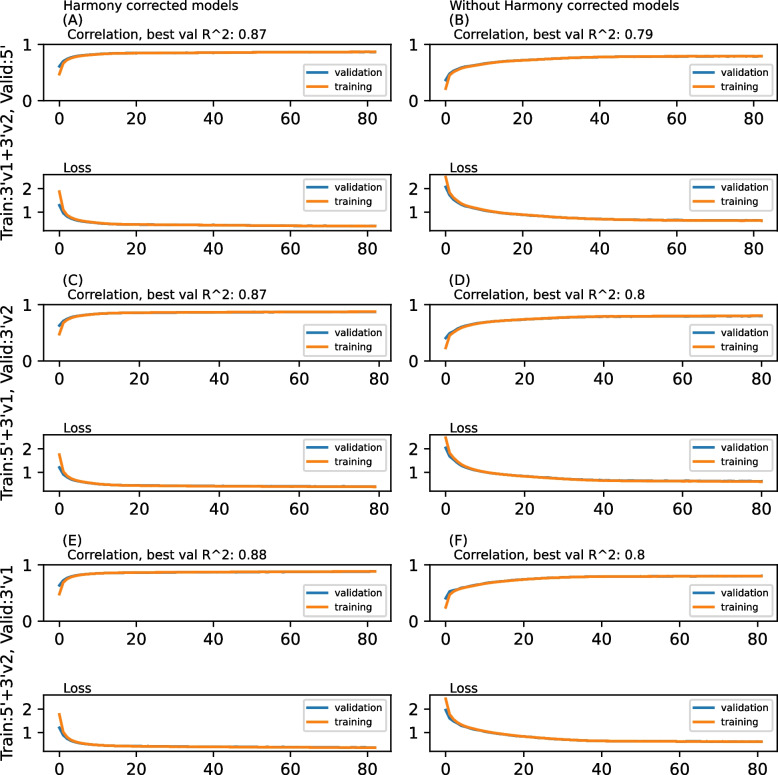
The curves of Pearson’s correlation coefficients and losses in the training of the HASCAD models. Each model was trained by using a set of 8000 simulated RNA-seq samples, and was validated by using another set of 8000 simulated RNA-seq samples. (**A**) and (**B**): The training data were simulated from the 3’v1 (threepv1) and 3’v2 (threepfresh) sets. The validation data were simulated from the 5’(fivePrime) set. (**C**) and (**D**): The training data were simulated from the 3’v1 (threepv1) and 5’ (fivePrime) sets. The validation data were simulated from the 3’v2 (threepfresh) set. (**E**) and (**F**): The training data were simulated from the 3’v2 (threepfresh) and 5’ (fivePrime) sets. The validation data were simulated from the 3’v1 (threepv1) set. In (**A**), (**C**), and (**E**), the scRNA-seq data were normalized by harmony. In (**B**), (**D**), and (**F**), the scRNA-seq data were not normalized by Harmony. The training was stopped when the model was not improved in loss function for the evaluation after 20 epochs

It was noted that all of the HASCAD models converged in approximately 80 epochs and no significant overfitting was noticed (Fig. 4). As what has been expected, the Harmony-corrected models showed better performances than the non-Harmony corrected models. In both the training and testing of the models, the result showed that there were higher Pearson’s correlation coefficients (PCCs) and lower losses in the training of Harmony-corrected models, compared to those of non-Harmony-corrected model (Fig. 4).

**Fig. 5:**
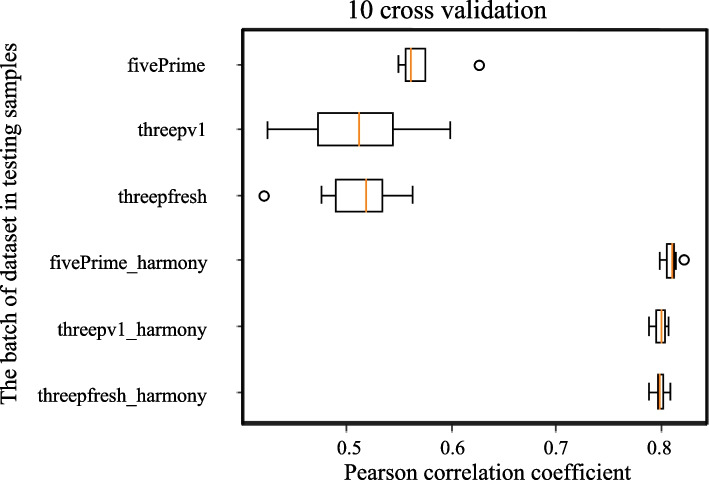
The Pearson’s correlation coefficients between the predicted cell fractions and the ground truths, using a tenfold cross validation approach

In addition, a 10-fold cross validation was conducted to further assess the performance of the HASCAD models. The result shows that, for the Harmony-corrected models, the Pearson’s correlation coefficients (PCCs) between the predicted cell fractions and the ground-truth were over 0.79, whereas the best PCC of all non-Harmony-corrected models was 0.63 (Fig. 5). This result suggests that the batch variation in the training data was likely to cause overfitting in model training. By contrast, the models trained by using the simulated bulk RNA-seq data based on the batch-variation removed scRNA-seq data were more robust.

**Fig. 6:**
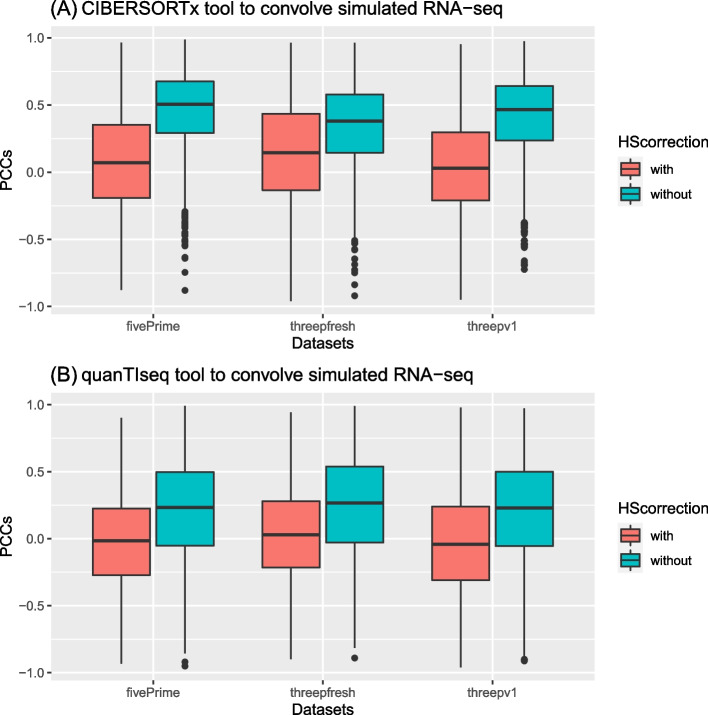
The Pearson’s correlation coefficients between the predicted cell fractions and the ground truths from gene expression with/without Harmony-Symphony correction, which were analyzed (**A**) CIBEROSRTx and (**B**) quanTIseq

In addition, we also assessed if CIBERSORTx and quanTIseq could predict the cell fractions in the simulated bulk RNA-seq data that were generated using the batch variation-removed scRNA-seq datasets. To follow the usage suggestions of these two tools, we prepared the input data by transforming the simulated data of bulk RNA-seq to TPM values. It turns out that by using CIBERSORTx and quanTIseq the PCCs of the predictions with the ground truths were better when the simulated bulk RNA-seq data were derived from non H–S corrected scRNA-seq data (Fig. 6A for the CIBERSORTx predictions, and Fig. 6B for the quanTIseq predictions).

## Benchmarks

### The benchmark using the RNA-seq of pure-type immune cells

To compare the performance of HASCAD with those of other CCD methods, we started by using the bulk RNA-seq data of pure-type immune cells. NCBI GEO GSE141498 was used in this benchmark, and the dataset includes 49 samples of B cells, 61 samples of DC cells, and 50 samples of monocytes. However, one consideration is that there is no information about the fractions of immune cell subtypes in this dataset. Besides, the cell subtypes that can be analyzed by different CCD methods are not perfectly the same. Therefore, we created the mapping between the ground-truth cell types in GSE141498 to the immune cell types that could be predicted by the CCD methods (Table 4). For example, HASCAD can predict the cell fractions of naïve and memory B cells, and their sum was regarded as the prediction fraction for B cells.

**Table 4:** Mapping the ground-truth cell types in GSE141498 to the immune cell types predicted by different CCD methods

	HASCAD	CIBERSORTx	quanTIseq
B cells	bnaive bmem	B.cells.naive B.cells.memory	B.cells
CD4 T cells	cd4mem cd4naive treg	T.cells.CD4.naive T.cells.CD4.memory.resting T.cells.CD4.memory.activated T.cells.regulatory..Tregs	T.cells.CD4 Tregs
Dendritic cells	pdc adc	Dendritic.cells.resting Dendritic.cells.activated	Dendritic.cells
Monocytes	mono14 mono16	monocytes	Monocytes

To estimate the accuracy of different CCD methods when analyzing the RNA-seq data consisting of pure-type immune cells, we took the highest predicted cell fraction to determine the major immune cell type. For example, in analyzing such a sample consisting of only one cell type, if the predicted fraction of cell type *X* is higher than those of the other cell types *Y*’s, then the major cell type of this sample would be assigned with *X*.

The result of the benchmark analyzing the pure-type immune cell samples is summarized in Table 5. It is noted that HASCAD achieved 100% accuracy for the four cell types in this benchmark. By contrast, CIBERSORTx did not perform well on the pure-type immune cell samples of DC cells; many pure DC cell samples were predicted as monocytes, and some pure DC cell samples were even predicted as CD8 T cells, B cells, NK cells, and Mas cells. QuanTIseq performed well on the pure-type immune cell samples of monocytes and CD4 T cells, but its accuracies were lowered to 45% and 52% for the cases of B cells and DC cells, respectively. Therefore, HASCAD outperforms CIBERSORTx and quanTIseq in the cell fraction prediction of RNA-seq samples, each consisting of only a pure type of immune cells.

**Table 5:** The accuracies of HASCAD, CIBERSORTx, and quanTIseq in analyzing the pure-type immune cell samples in NCBI GEO GSE141498

	B cells	CD4 T cells	Dendritic cells	Monocytes
HASCAD	100%	100%	100%	100%
CIBERSORTx	98%	96%	0%	98%
quanTIseq	45%	100%	52%	96%

### The benchmark using human bulk RNA-seq datasets

Before we assessed the performances of HASCAD and other two CCD methods by using the human PBMC RNA-seq dataset, NCBI GEO GSE107572, we first evaluated if a pre-processing of the input data by applying the Harmony-Symphony correction might benefit the task of CCD. To prepare the reference data to analyze this PBMC RNAseq set, VST was used to select genes shared by the three PBMC scRNA-seq datasets and GSE107572. Thus, a set of 1,158 VST filtered genes was used in this benchmark of HASCAD (Additional file 1: Figure S1).

**Fig. 7:**
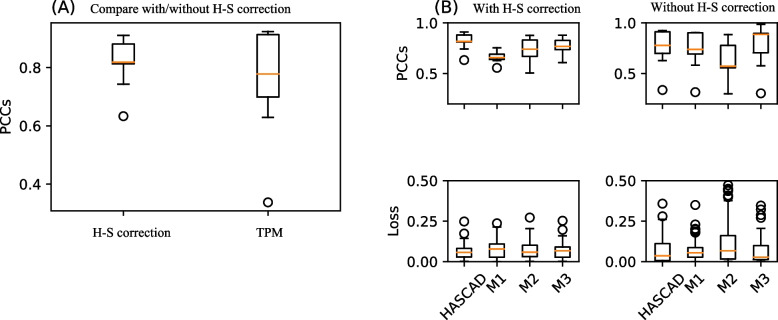
The performance of HASCAD assessed by using Symphony-corrected and non-Symphony corrected (TPM) data as the input to predict cell fractions. Pearson's correlation coefficients and mean squared errors between the predicted cell fractions and the ground truths were calculated. M1, M2, and M3 refer to the three subnetworks in the ensemble architecture of HASCAD model

Interestingly, our result suggests that HASCAD has a better performance when the input data has been normalized by applying the H–S correction approach (Fig. 7). When the input data has been pre-processed, the mean PCC of HASCAD was around 0.8 (Fig. 7A, "Symphony"), higher than that (mean PCC =  ~ 0.6) of the model in analyzing the non H–S corrected input data (Fig. 7A, "TPM"). In addition, there were a higher mean PCC and a lower MSE when the HASCAD model analyzed the H–S corrected input data (Fig. 7B, "With Symphony" versus "Without Symphony").

In addition, HASCAD model is an ensemble of three subnetworks, namely M1, M2, and M3 (Fig. 2), where the three sets of output values were averaged to give the predicted cell fractions. It was noted that the performance of each individual subnetwork was more varied than the ensemble model, and there were also higher MSEs and lower PCCs (Fig. 7B), no matter whether the input data has been normalized by using the H–S correction approach. This result suggests that the usage of the ensemble of the three subnetworks can improve the robustness of HASCAD to predict immune cell fractions in bulk RNA-seq samples.

In the final benchmark, we compared the performance of HASCAD with those of two widely used models, CIBERSORTx and quanTIseq, in predicting the fractions of immune cells of real tissue samples. Hence, nine bulk RNA-seq datasets of human PBMC samples, GSM2871599-GSM2871607, were downloaded from NCBI GEO. The fractions of seven cell types in these datasets have been experimentally determined, and the cell-type mapping between the predictions and the ground-truth is listed in Table 6.

**Table 6:** Cell-type mapping of CIBERSORTx, quanTIseq, and HASCAD to the cell types investigated in GSE107572

GSE107572	CIBERSORTx	HASCAD	quanTIseq
NK	NK cells resting NK cells activated	NK	NK.cell
B cell	B.vaive	bnaive, bmem	B.cells
DC	Dendritic cells resting Dendritic cells activated	pdc, adc	Dendritic.cells
monocyte	monocyte	mono14, mono16	Monocytes
CD8	T.cells.CD8	dd8naive, cd8eff, cd8mem	T.cells.CD8
CD4	T.cells.CD4.naïve	c4naive, cd4mem	T.cells.CD4
Treg	T.cells.regulatory..Tregs	treg	Tregs
neutrophils	X	X	X
X	X	hsc, mk	X

X: no this cell type in the resource

**Fig. 8:**
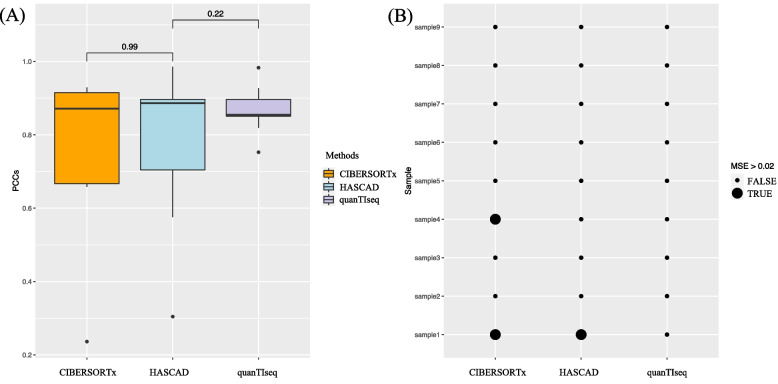
(**A**) The Pearson’s correlation coefficients and (**B**) The mean square errors (MSE) of the predicted cell fractions made by CIBERSORTx, HASCAD, and quanTIseq, by analyzing the nine human PBMC RNA-seq datasets. A bigger blackish dot is to indicate a MSE greater than 0.02

From the distributions of Pearson’s correlation coefficients (Fig. 8A), the correlation between the ground truth and predictions made by HASCAD might be better, though the *p*-value was not significant due to the small sample size. As mean square error (MSE) was used as the performance metrics, it is obvious that the performance of CIBERSORTx was worse than those of HASCAD and quanTIseq (Fig. 8B). The result suggests that the performance of HASCAD is at least comparable to that of quanTIseq, and HASCAD is able to predict the cell fractions of six additional cell types.

In addition, in three out of the nine samples the PCCs of CIBERSORTx predictions were lower than 0.7, and the lowest value was only 0.24. By contrast, by using HASCAD, all of the PCCs of the HASCAD predictions were higher than 0.6 (Additional file 1: Figure S2—Figure S4). The standard deviations (SDs) of the prediction-ground truth differences for HASCAD, CIBERSORTx, and quanTIseq were 0.07, 0.12, and 0.08, respectively, and the mean and SD values for each cell type are presented in Bland–Altman plots (BA plots, Additional file 1: Figure S5—Figure S7).

By using the same datasets of the nine human PBMC bulk RNA-seq samples, we additionally assessed xCell (Additional file 1: Table S1), which is a CCD method based on ssGSEA. It turns out that all of the PCCs of xCell predictions were lower than 0.23, and some were even negative values (Additional file 1: Figure S8), suggesting that xCell is unsuitable for the deconvolution of these human PBMC bulk RNA-seq samples.

Survival analysis of TCGA-LIHC samples with HASCAD-predicted high/low immune cell abundances.

**Fig. 9:**
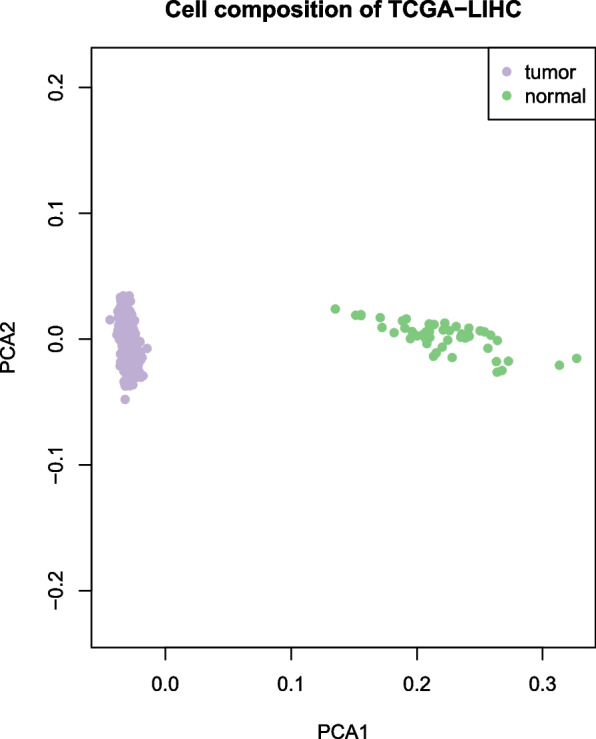
A PCA plot showing distinct clustering of 50 normal samples and 364 tumor samples of TCGA-LIHC, using the cell compositions predicted by HASCAD as the features

By applying our HASCAD model trained on the H–S normalized data of three PBMC scRNA-seq datasets, we investigated the association of cancer patients’ survival with the predicted immune cell abundance. The gene expression data of the bulk RNA-seq of 364 TCGA-LIHC patients and 50 normal tissue samples, and their clinical data were downloaded from NCI Genomic Data Commons (NCI GDC, https://gdc.cancer.gov/). Using the HASCAD predicted cell proportions as the features, the distribution of the samples was visualized by PCA. Clearly there are two distinct clusters, the normal cluster and the tumor cluster, suggesting that the immune cell compositions of tumor samples are very different from those of normal samples (Fig. 9).

After excluding the ones that do not have the records of overall survival status, 359 bulk tumor RNA-seq samples remained for the survival analysis (Additional file 1: Figure S10). The results of the survival analysis revealed that there is a positive association of the HASCAD-predicted abundance of effector memory CD8 T cells and memory CD8 T cells with TCGA-LIHC patients’ prognosis (Additional file 1: Figure S11, S12). On the other hand, there is a negative association of the HASCAD-predicted abundance of naïve CD8 T cells and hematopoietic stem cells with patients’ prognosis (Additional file 1: Figure S13, S14).

**Fig. 10:**
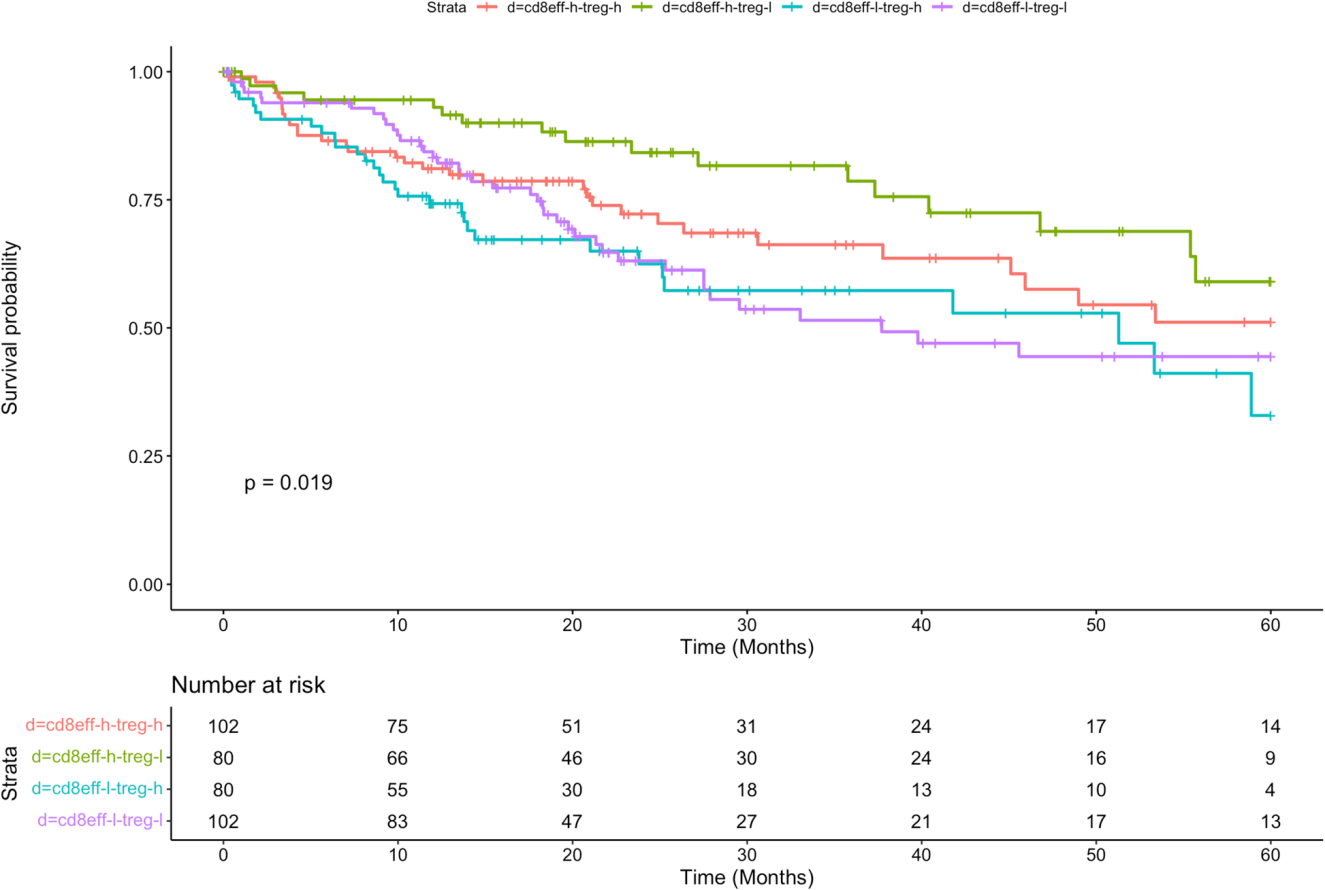
A Kaplan–Meier curve plot showing overall survival for four immune-abundance groups from TCGA-LIHC by HASCAD (log-rank test, *p*-value = 0.019). High Effector CD8 T cells-High Treg (cd8eff-h-treg-h), High Effector CD8 T cells-Low Treg (cd8eff-h-treg-l), Low Effector CD8 T cells-High Treg, (cd8eff-l-treg-h) and Low Effector CD8 T cells-Low Treg (cd8eff-l-treg-l)

Effector CD8 T cells is essential in the anti-tumor immune response, whereas regulatory T cells (Treg) are involved in tumor development and progression by inhibiting antitumor immunity [23]. To explore the association of the abundance of these two cell types with TCGA-LIHC patients’ prognosis, we divided the patients into four groups: 1) High effector CD8 T cells-High Treg; 2) High effector CD8 T cells-Low Treg; 3) Low effector CD8 T cells-High Treg; 4) Low effector CD8 T cells-Low Treg. The result of the log rank test revealed that there was significant difference in the overall survival time of the four patient groups (*p* value = 0.019, Fig. 10). The group that has the best overall survival time is High effector CD8 T cells-low Treg (Fig. 10, the green line), and the group with the worse survival is “Low effector CD8 T cells-High Treg” (Fig. 10, the blue line).

## Discussion

HASCAD is a cell composition deconvolution (CCD) method we designed to predict the immune cell fractions in bulk RNA-seq samples. HASCAD has been implemented by using a deep neural network architecture, and it has been trained by using the reference cell-specific gene expression signatures derived from scRNA-seq data. Unlike regression-based CCD methods that have assumed a linear relation between bulk gene expression profiles and the fractions of various cell types, HASCAD takes advantage of the non-linear property of neural network, which might improve the robustness for the prediction of immune cell fractions. Compared to a previous DNN model designed for CCD, Scaden, which could analyze only five immune cell types, HASCAD could predict the cell fractions of as many as 15 immune cell types. To reduce the overfitting that might be caused by using scRNA-seq data containing batch variations, the training and validation data for optimizing HASCAD have been prepared by applying a batch-effect removal approach, the Harmony-Symphony correction. We note that by using the training data in which the batch variations have been removed to a certain extent, HASCAD is likely to have robust performance. Besides, the performance of HASCAD might be further improved if the input RNA-seq data could be pre-processed by using the Harmony-Symphony correction strategy.

In the benchmark using human PBMC RNA-seq datasets, the performance of HASCAD is similar to that of quanTIseq, better than CIBERSORTx. HASCAD is superior to quanTIseq in that it can prediction the fractions of more cell types. With the fast and wide application of the scRNA-seq technology in various studies, it can be expected that there will be more scRNA-seq datasets that can be used to optimize CCD methods in the future. HASCAD, a DNN based method, could be readily tuned by using new datasets.

On the other hand, we notice that analyzing the RNA-seq sample with a highly skewed distribution of cell fractions can be very challenging to CCD methods. For example, HASCAD usually gave a predicted cell fraction very deviated from 100% when analyzing the RNA-seq samples consisting of only the pure-type immune cells. Other two CCD methods, CIBERSORTx and quanTIseq also have such a limitation. We therefore adopted an alternative approach to assess the three methods, where the cell type predicted to have the highest fraction value was assigned as the major cell type of such pure-type RNA-seq samples. In the 209 pure-type RNA-seq samples, HASCAD is able to correctly identify the major type of immune cells, whereas CIBERSORTx and quanTIseq does not perform well in the cases of at least one cell types.

We used HASCAD to estimate the immune cell compositions in TAGA-LIHC samples, and performed survival analysis to explore the association between immune cell abundance and patients’ prognosis. Interestingly, the log-rank tests revealed significant associations with patients’ overall survival in the immune-cell abundance groups of hematopoietic stem cells (HSC) and three subtypes of CD8 T cells (Additional file 1: Figure S11-S14). Our finding is consistent with those of previous studies about the type-specific impact of immune cells on cancer prognosis. For example, Lu *et. al.* reported that glioblastoma patients with higher levels of HSCs had poor prognosis [24]. Consistently, we found a negative relationship between survival and HSC abundance in liver cancer. In addition, our result is consistent with the findings supporting that Treg cells can suppress the anti-tumor function of effect CD8 T cells, and thus Treg cells can promote the growth of cancer cells [25].

Tumor is a complex ecosystem and TME consists of various cell types. Members of each cell type might be further regulated to differentiate into functionally distinct cell subtypes due to their varied positioning in TME. Although scRNA-seq and spatial transcriptomics are able to provide a more comprehensive view of TME, in large-scale cancer studies they are usually not the first choice for analyzing the transcriptomic changes of many samples due to their high costs. Alternatively, using scRNA-seq datasets as the reference to perform cell type deconvolution of bulk tissue RNA-seq is likely to be a more affordable approach to explore the cell compositions in TME. To cover the diverse cell types in TME in CCD, one plausible approach is to integrate multiple scRNA-seq datasets as the reference. In this study, we demonstrate that, on a limited scale, the data pre-processing strategy, Harmony-Symphony correction, can be applied to integrate three scRNA-seq datasets in the training of a CCD method. A future direction is to integrate more scRNA-seq datasets that might consist of different cell types, which might be derived from different tissue sources and studies, in order to create a more robust CCD method. Therefore, we expect that pre-processing strategies of scRNA-seq data that can remove technical biases and batch effects could play important roles in the future improvement of CCD methods.

## Conclusion

We developed a new deep neural network model, HASCAD, to perform the task of cell composition deconvolution (CCD) of bulk RNA-seq data. HASCAD has been trained by using the synthetic bulk RNA-seq data that were simulated by using three scRNA-seq datasets consisting of 15 immune cell types. To mitigate the batch-specific variations that may cause model overfitting, the scRNA-seq datasets are preprocessed by using a novel normalization approach, the Harmony-Symphony correction. We show that, in the benchmarks, the HASCAD model that is trained based on the Harmony-Symphony normalized scRNA-seq datasets can really achieve a better cross-dataset performance. HASCAD has a performance that is at least comparable to those of two widely used CCD methods, and it can predict more cell types than the other methods that were built based on the RNA-seq or scRNA-seq data.

### Supplementary Information

**Additional file 1:** **Figure S1.** Venn diagram showing the number of genes in the reference and GSE data. **Figure S2.** The scatter plots of the ground-truth and predicted cell abundance made by HASCAD for the nine human PBMC bulk RNA-seq samples. Each point corresponds to a cell type in each sample. R^2 refers to Pearson’s correlation coefficient (PCC). **Figure S3.** The scatter plots of the ground-truth and cell abundance predictions made by CIBERSORTx for the nine human PBMC bulk RNA-seq samples. Each point corresponds to a cell type in each sample. R^2 refers to Pearson’s correlation coefficient (PCC). **Figure S4.** The scatter plots of the ground-truth and cell abundance predictions made by quanTIseq for the nine human PBMC bulk RNA-seq samples. Each point corresponds to a cell type in each sample. R^2 refers to Pearson’s correlation coefficient (PCC). **Figure S5.** The Bland-Altman plots (BA plots) of the cell type-specific differences between the ground- truth and the predictions made by HASCAD for the nine PBMC bulk RNA-seq samples. Each point corresponds to one of the nine PBMC bulk RNA-seq samples. **Figure S6.** The BA plots of the cell type-specific differences between the ground-truth and the predictions made by CIBERSORTx for the nine PBMC bulk RNA-seq samples. Each point corresponds to one of the nine PBMC bulk RNA-seq samples. **Figure S7.** The BA plots of the cell type-specific differences between the ground-truth and the predictions made by quanTIseq for the nine PBMC bulk RNA-seq samples. Each point corresponds to one of the nine PBMC bulk RNA-seq samples. **Figure S8.** The scatter plots of the ground-truth and cell abundance predictions made by xCell for the nine human PBMC bulk RNA-seq samples. R^2 refers to Pearson’s correlation coefficient (PCC). Each point corresponds to one of the nine PBMC bulk RNA-seq samples. **Figure S9.** The Venn diagram showing the number of genes reference scRNA-seq data and TCGA-LIHC bulk RNA-seq data. **Figure S10.** The barplot showing HASCAD-predicted proportions of various cell types in 364 TCGA- LIHC bulk RNA-seq samples. **Figure S11.** A Kaplan–Meier plot showing the difference in overall survival of TCGA-LIHC patients between the high and the low groups of HASCAD-predicted proportions of effector CD8 T cells. **Figure S12.** A Kaplan–Meier plot showing the difference in overall survival of TCGA-LIHC patients between the high and the low groups of HASCAD-predicted proportions of memory CD8 T cells. **Figure S13.** A Kaplan–Meier plot showing the difference in overall survival of TCGA-LIHC patients between the high and the low groups of HASCAD-predicted naïve CD8 T cells. **Figure S14.** A Kaplan–Meier plot showing the difference in overall survival of TCGA-LIHC patients between the high and the low groups of HASCAD-predicted proportions of hematopoietic stem cells. **Table S1.** Cell-type mapping of xCell to the cell types investigated in GSE107572.

## Data Availability

The datasets of this article were downloaded from the GEO database. GSE141498 is downloaded from https://www.ncbi.nlm.nih.gov/geo/query/acc.cgi?acc = GSE141498. GSE107572 is downloaded from https://www.ncbi.nlm.nih.gov/geo/query/acc.cgi?acc=GSE107572. PBMC scRNA-seq datasets is downloaded from https://github.com/immunogenomics/symphony.

## Abbreviations

HASCAD: HArmonized single-cell RNA-seq Cell type Assisted Deconvolution
CCD: Cell composition deconvolution
DNN: Deep neural network
TIICs: Tumor-infiltrating immune cells
M1: Classically activated macrophages
M2: Activated macrophages
Tregs: Regulatory T cells
IHC: Immune-histochemistry
aDC: Activated dendritic cell
pDC: Plasmacytoid dendritic cell
bmem: Memory B cells
bnaive: Naïve B cells
cd4mem: Memory CD4 T cells
cd4naive: Naïve CD4 T cells
cd8eff: Effector CD8 T cells
cd8mem: Memory CD8 T cells
cd8naive: Naïve CD8 T cells
HSC: Hematopoietic stem cell
MK: Megakaryocyte
mono14: CD14 monocyte
mono16: CD16 monocyte
NK: Nature killer cells
RNA-seq: RNA sequencing
GEP: Gene expression profiles
TME: Tumor microenvironment
refGEP: Reference GEP
ssGSEA: Single-sample gene set enrichment analysis
PBMC: Peripheral blood mononuclear cell
PCA: Principal component analysis
VST: Variance stabilizing transform
TPM: Transcripts-per-millions
MSE: Mean square error
PCC: Pearson’s correlation coefficient
H–S correction: Harmony-Symphony correction
TCGA-LIHC: The Cancer Genome Atlas Liver Hepatocellular Carcinoma
GDC: Genomic Data Commons
SDs: Standard deviations
