# Supplementary material for: HArmonized single-cell RNA-seq Cell type Assisted Deconvolution (HASCAD)
Source: BMC Med Genomics. 2023 Oct 31;16(Suppl 2):272. doi: 10.1186/s12920-023-01674-w (PMC10619225; doi:10.1186/s12920-023-01674-w)
Supplement: Supplementary file 1 — Additional file 1: Figure S1. Venn diagram showing the number of genes in the reference and GSE data. Figure S2. The scatter plots of the ground-truth and predicted cell abundance made by HASCAD for the nine human PBMC bulk RNA-seq samples. Each point corresponds to a cell type in each sample. R^2 refers to Pearson’s correlation coefficient (PCC). Figure S3. The scatter plots of the ground-truth and cell abundance predictions made by CIBERSORTx for the nine human PBMC bulk RNA-seq samples. Each point corresponds to a cell type in each sample. R^2 refers to Pearson’s correlation coefficient (PCC). Figure S4. The scatter plots of the ground-truth and cell abundance predictions made by quanTIseq for the nine human PBMC bulk RNA-seq samples. Each point corresponds to a cell type in each sample. R^2 refers to Pearson’s correlation coefficient (PCC). Figure S5. The Bland-Altman plots (BA plots) of the cell type-specific differences between the ground- truth and the predictions made by HASCAD for the nine PBMC bulk RNA-seq samples. Each point corresponds to one of the nine PBMC bulk RNA-seq samples. Figure S6. The BA plots of the cell type-specific differences between the ground-truth and the predictions made by CIBERSORTx for the nine PBMC bulk RNA-seq samples. Each point corresponds to one of the nine PBMC bulk RNA-seq samples. Figure S7. The BA plots of the cell type-specific differences between the ground-truth and the predictions made by quanTIseq for the nine PBMC bulk RNA-seq samples. Each point corresponds to one of the nine PBMC bulk RNA-seq samples. Figure S8. The scatter plots of the ground-truth and cell abundance predictions made by xCell for the nine human PBMC bulk RNA-seq samples. R^2 refers to Pearson’s correlation coefficient (PCC). Each point corresponds to one of the nine PBMC bulk RNA-seq samples. Figure S9. The Venn diagram showing the number of genes reference scRNA-seq data and TCGA-LIHC bulk RNA-seq data. Figure S10. The barplot showing HASCAD-pred [file 12920_2023_1674_MOESM1_ESM.pdf]

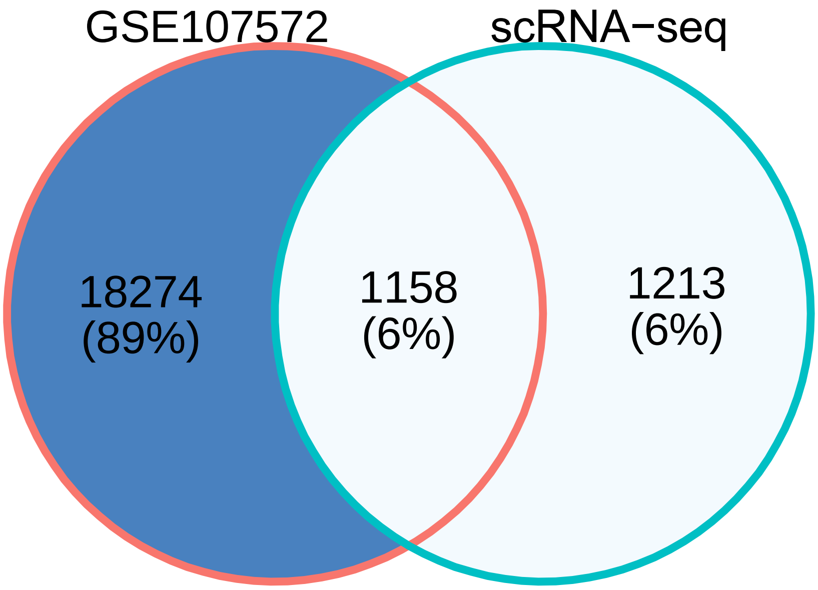


*Figure S1. Venn diagram showing the number of genes in the reference and GSE data.*

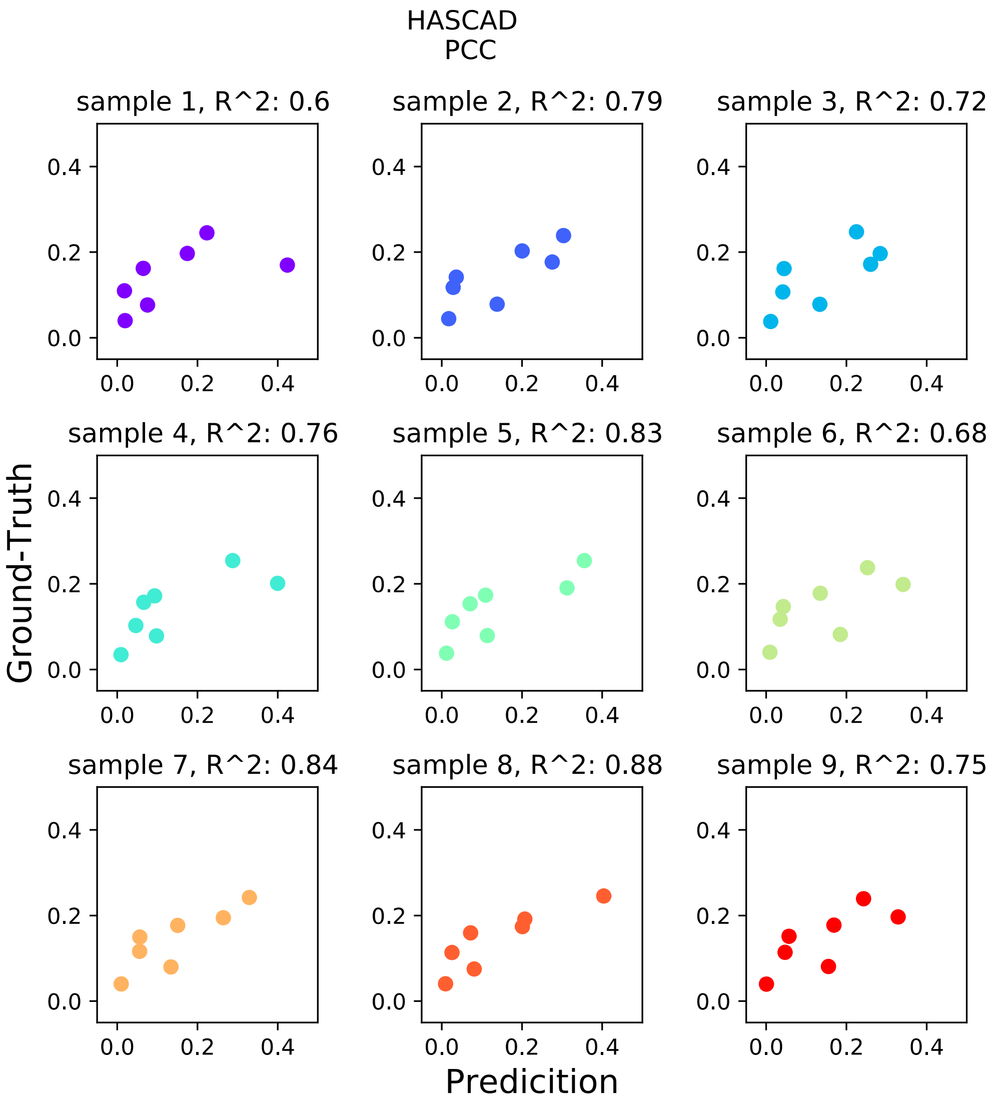


*Figure S2. The scatter plots of the ground-truth and predicted cell abundance made by HASCAD for the nine human PBMC bulk RNA-seq samples. Each point corresponds to a cell type in each sample. R^2 refers to Pearson's correlation coefficient (PCC).*

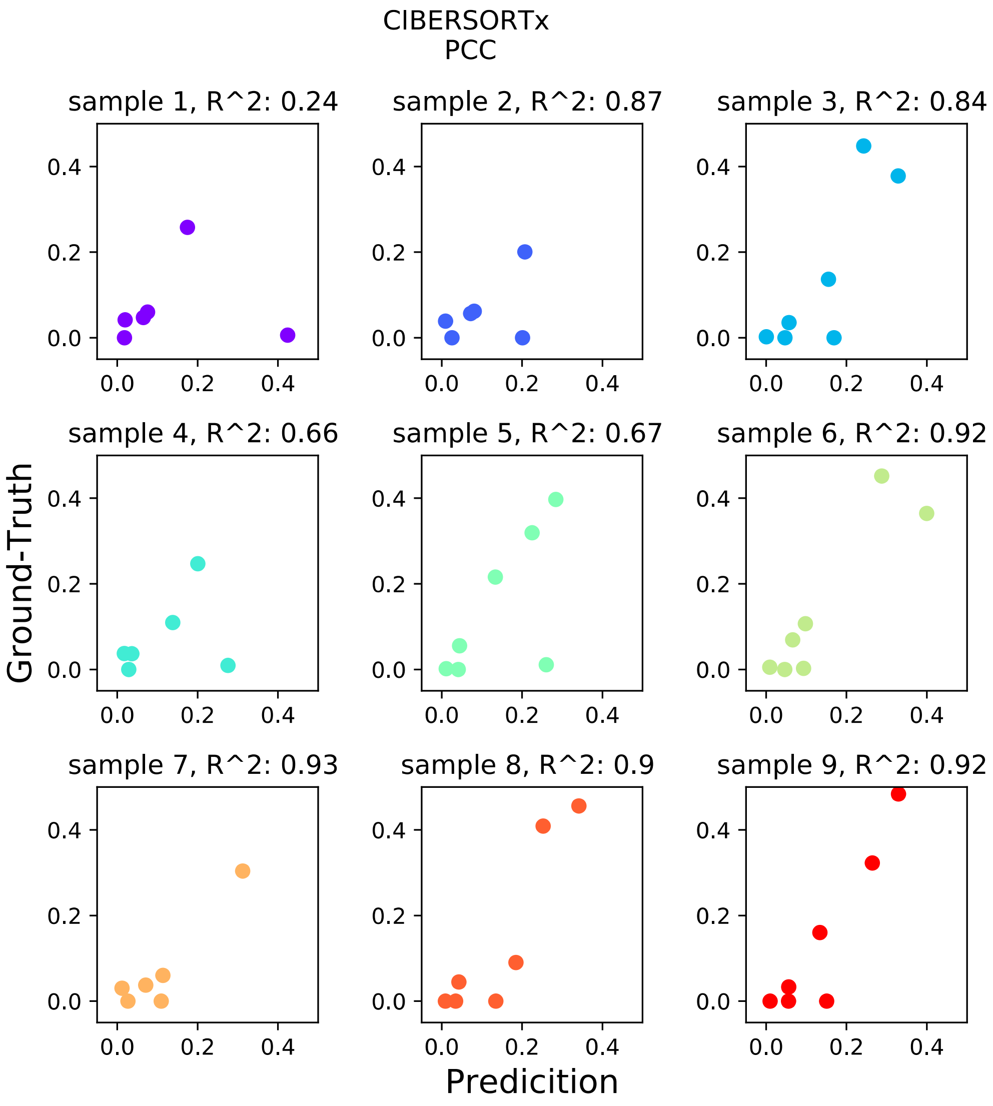


*Figure S3. The scatter plots of the ground-truth and cell abundance predictions made by CIBERSORTx for the nine human PBMC bulk RNA-seq samples. Each point corresponds to a cell type in each sample. R^2 refers to Pearson's correlation coefficient (PCC).*

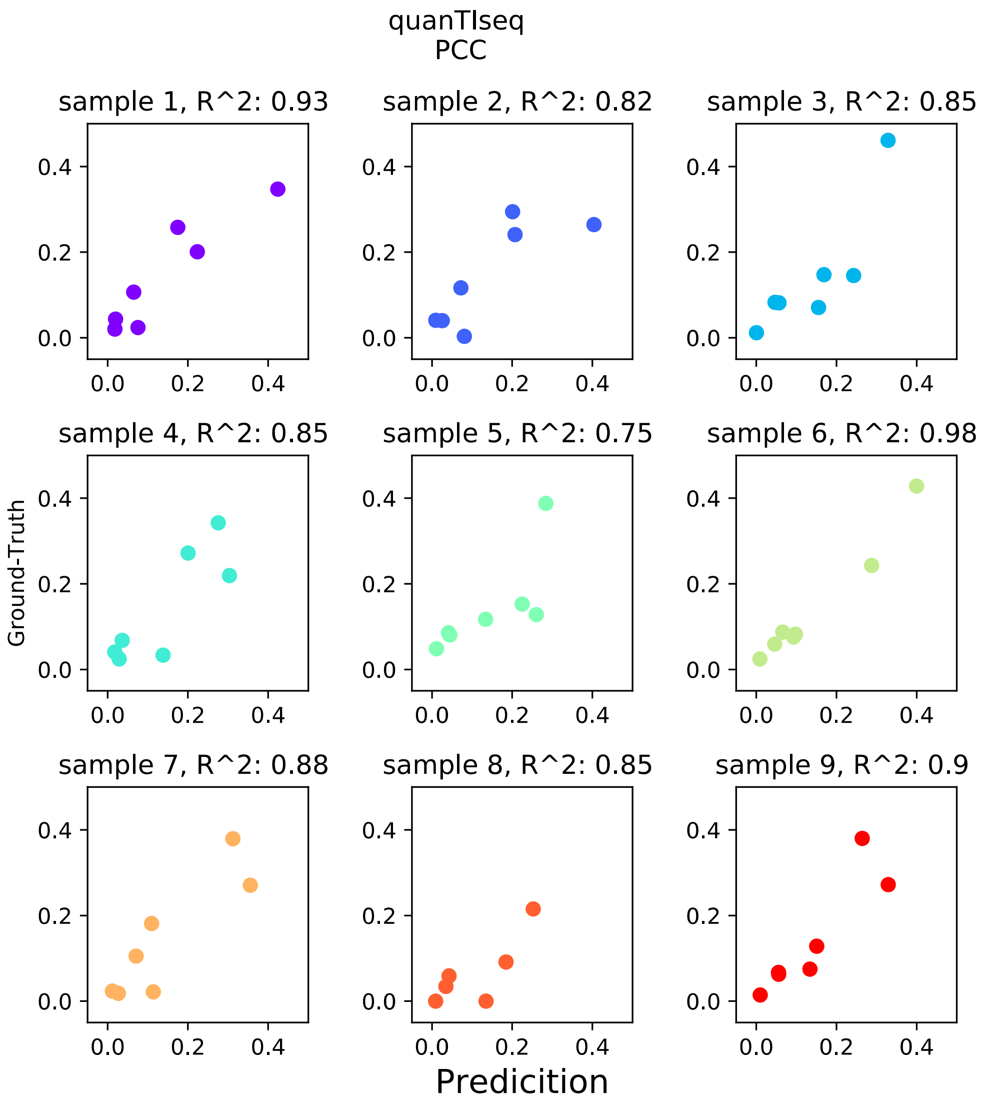


*Figure S4. The scatter plots of the ground-truth and cell abundance predictions made by quanTIseq for the nine human PBMC bulk RNA-seq samples. Each point corresponds to a cell type in each sample. R^2 refers to Pearson's correlation coefficient (PCC).*

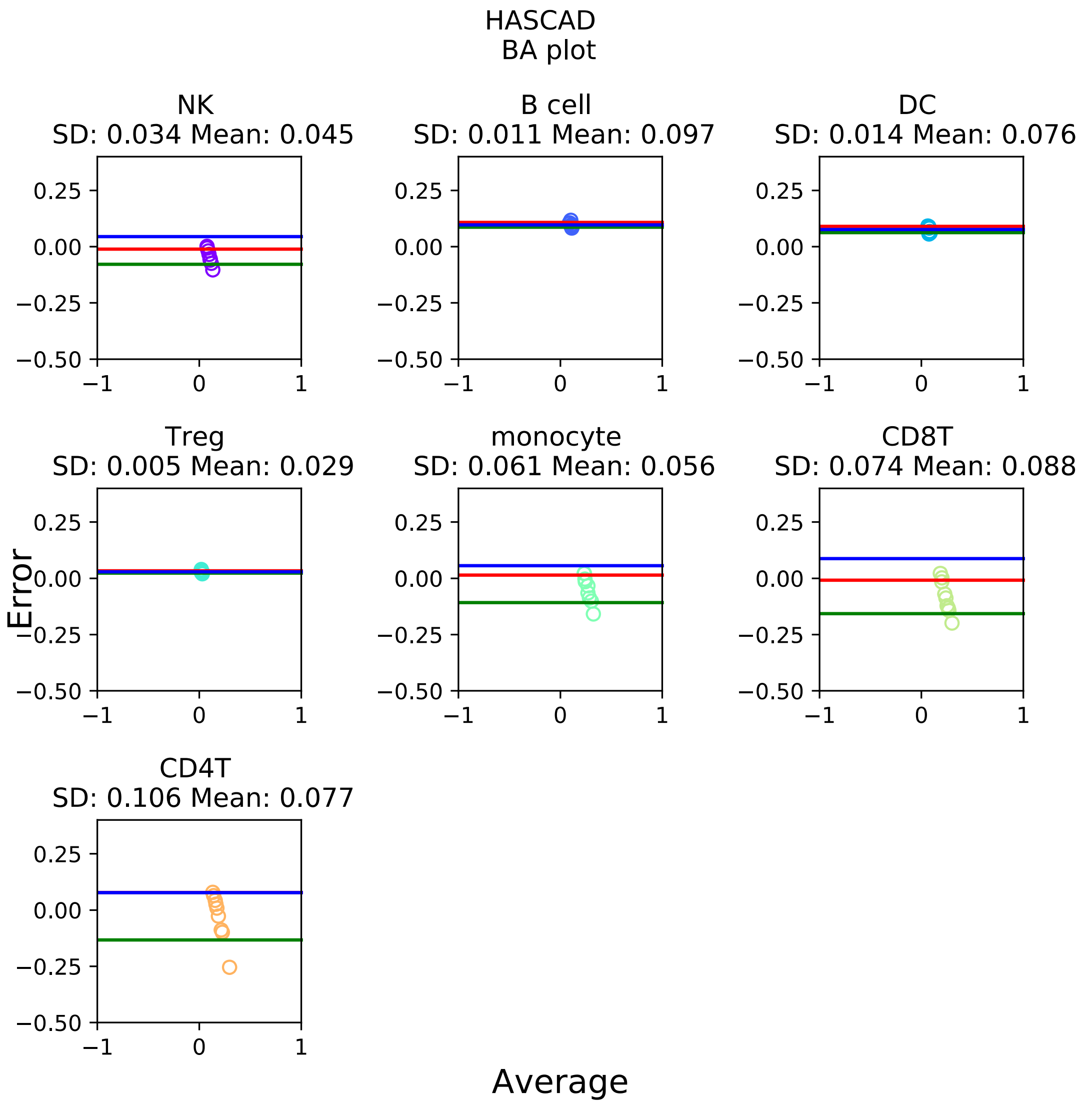


*Figure S5. The Bland-Altman plots (BA plots) of the cell type-specific differences between the ground-truth and the predictions made by HASCAD for the nine PBMC bulk RNA-seq samples. Each point corresponds to one of the nine PBMC bulk RNA-seq samples.*

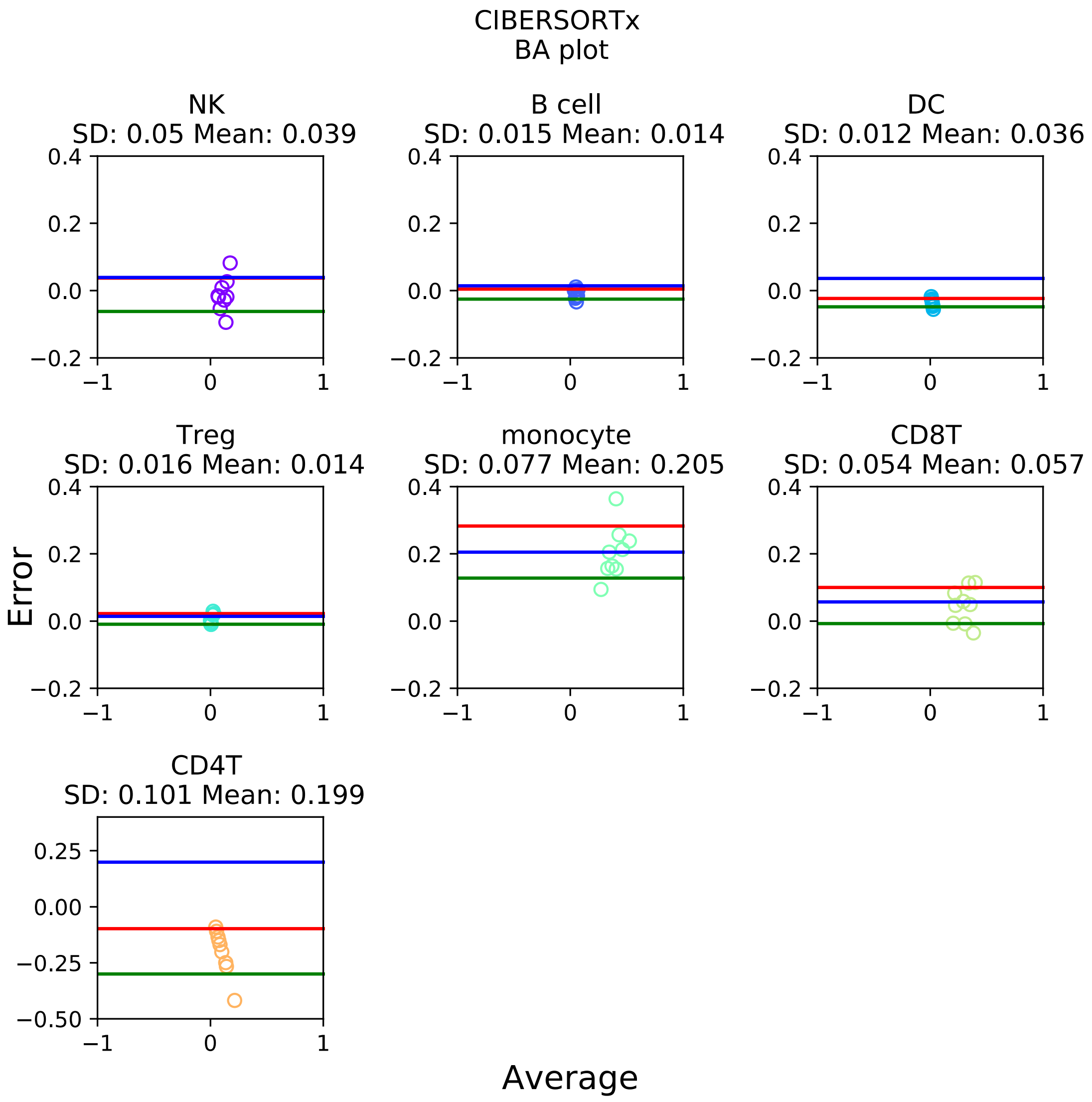


*Figure S6. The BA plots of the cell type-specific differences between the ground-truth and the predictions made by CIBERSORTx for the nine PBMC bulk RNA-seq samples. Each point corresponds to one of the nine PBMC bulk RNA-seq samples.*

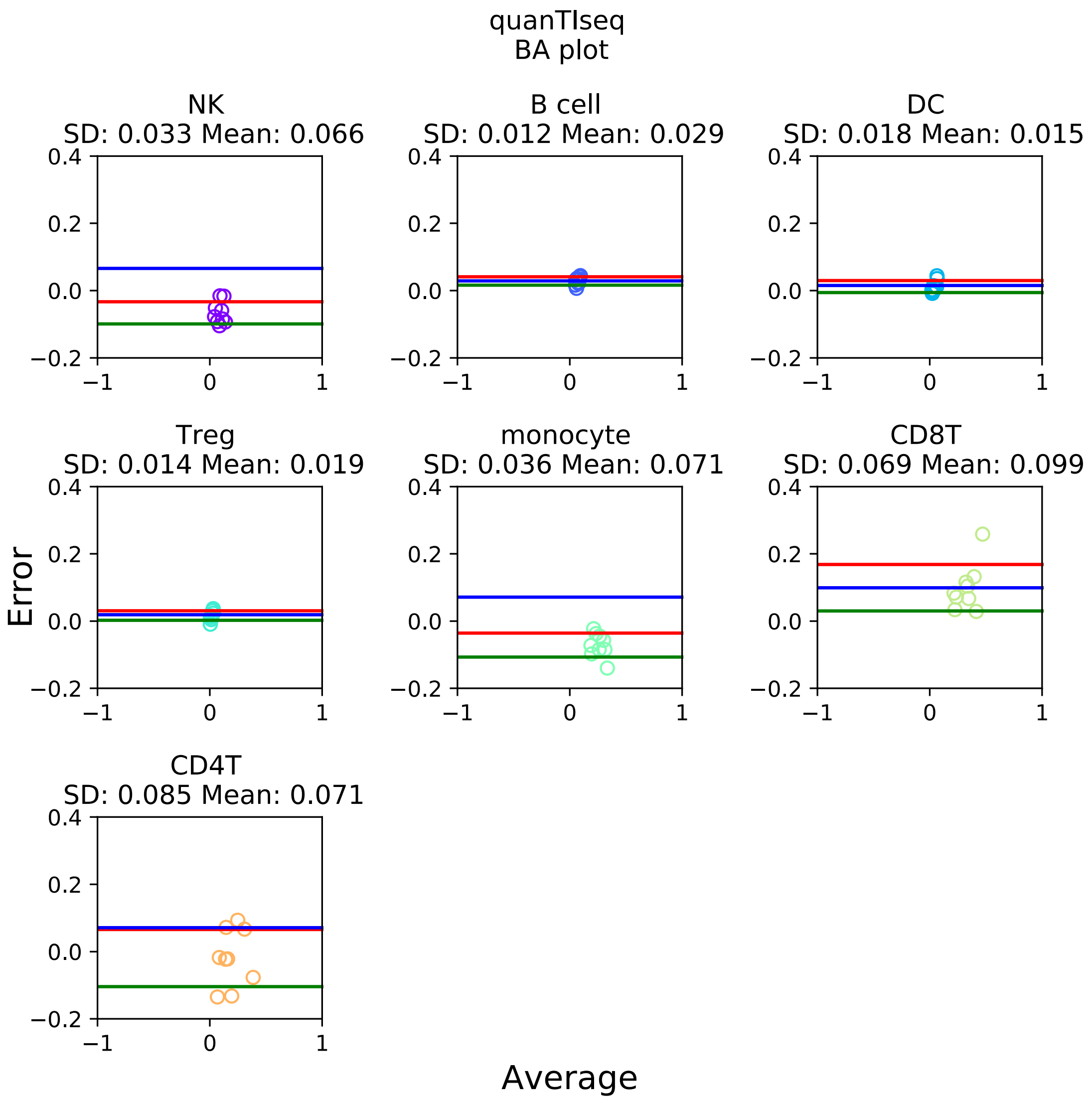


*Figure S7. The BA plots of the cell type-specific differences between the ground-truth and the predictions made by quanTIseq for the nine PBMC bulk RNA-seq samples. Each point corresponds to one of the nine PBMC bulk RNA-seq samples.*

*Table S1 Cell-type mapping of xCell to the cell types investigated in GSE107572*

| ***GSE107572*** | ***Xcell*** |
|---|---|
| *NK* | *NK cells* |
| *B cell* | *B-cells*<br>*naive B-cells*<br>*Memory B-cells*<br>*pro B-cells'* |
| *DC* | *aDC*<br>*cDC*<br>*iDC*<br>*pDC*<br>*DC* |
| *monocyte* | *Monocytes* |
| *CD8* | *CD8+ T-cells*<br>*CD8+ Tcm*<br>*CD8+ Tem*<br>*CD8+ naive T-cells* |
| *CD4* | *CD4+ T-cells*<br>*CD4+ Tcm*<br>*CD4+ Tem*<br>*CD4+ memory T-cells*<br>*CD4+ naive T-cells* |
| *Treg* | *Tregs* |
| *neutrophils* | *Neutrophils* |

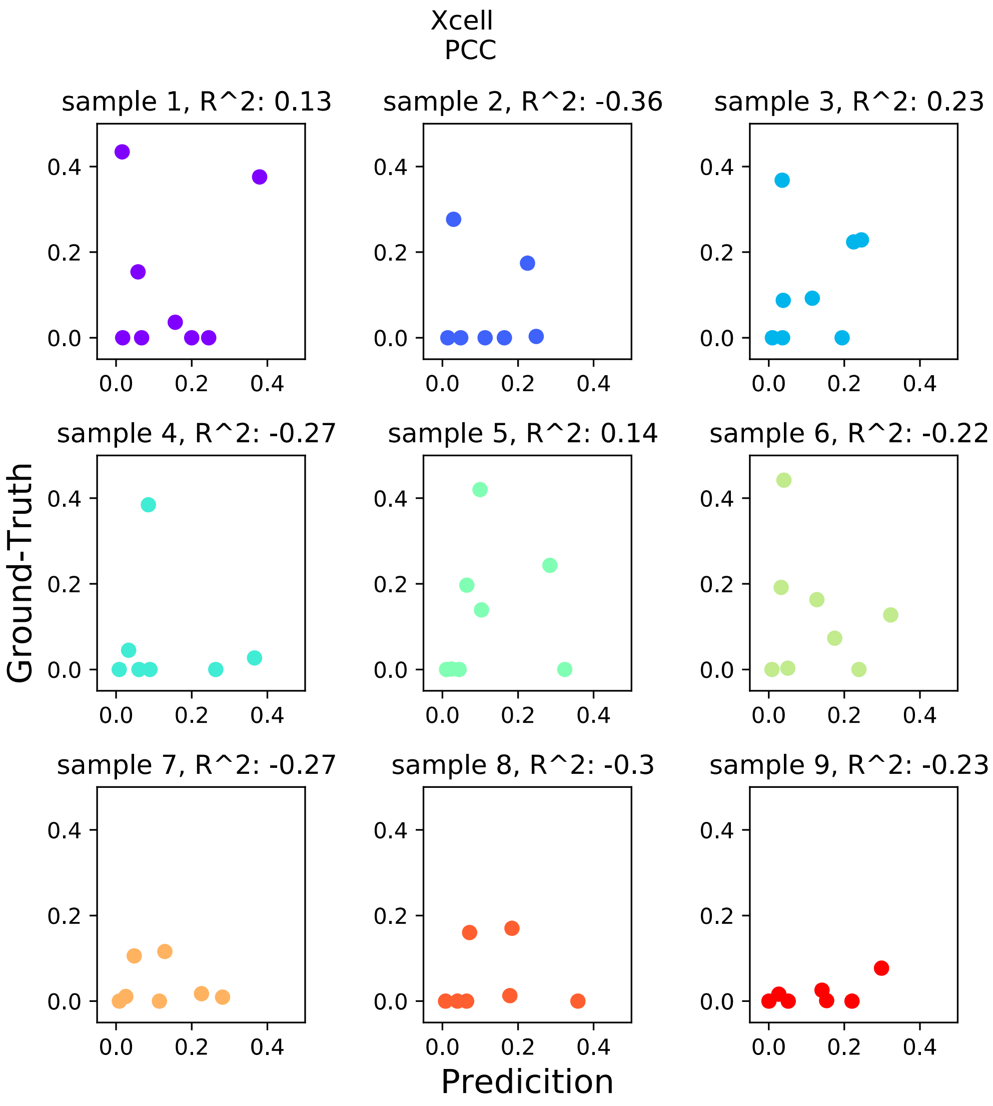


*Figure S8. The scatter plots of the ground-truth and cell abundance predictions made by xCell for the nine human PBMC bulk RNA-seq samples. R^2 refers to Pearson's correlation coefficient (PCC). Each point corresponds to one of the nine PBMC bulk RNA-seq samples.*

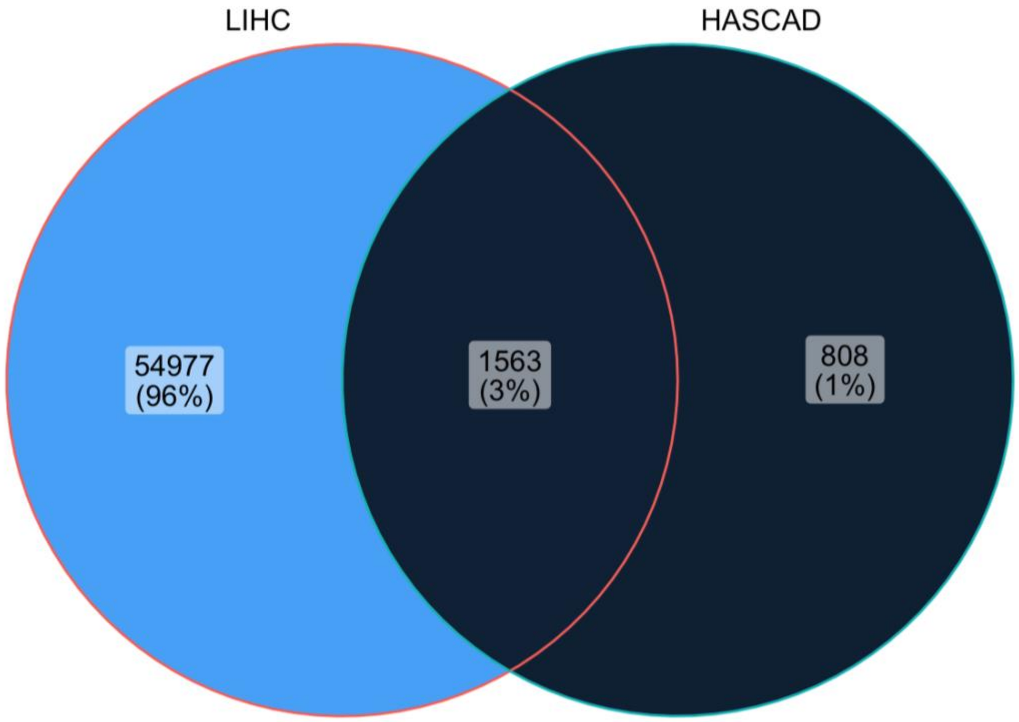


*Figure S9. The Venn diagram showing the number of genes reference scRNA-seq data and TCGA-LIHC bulk RNA-seq data.*

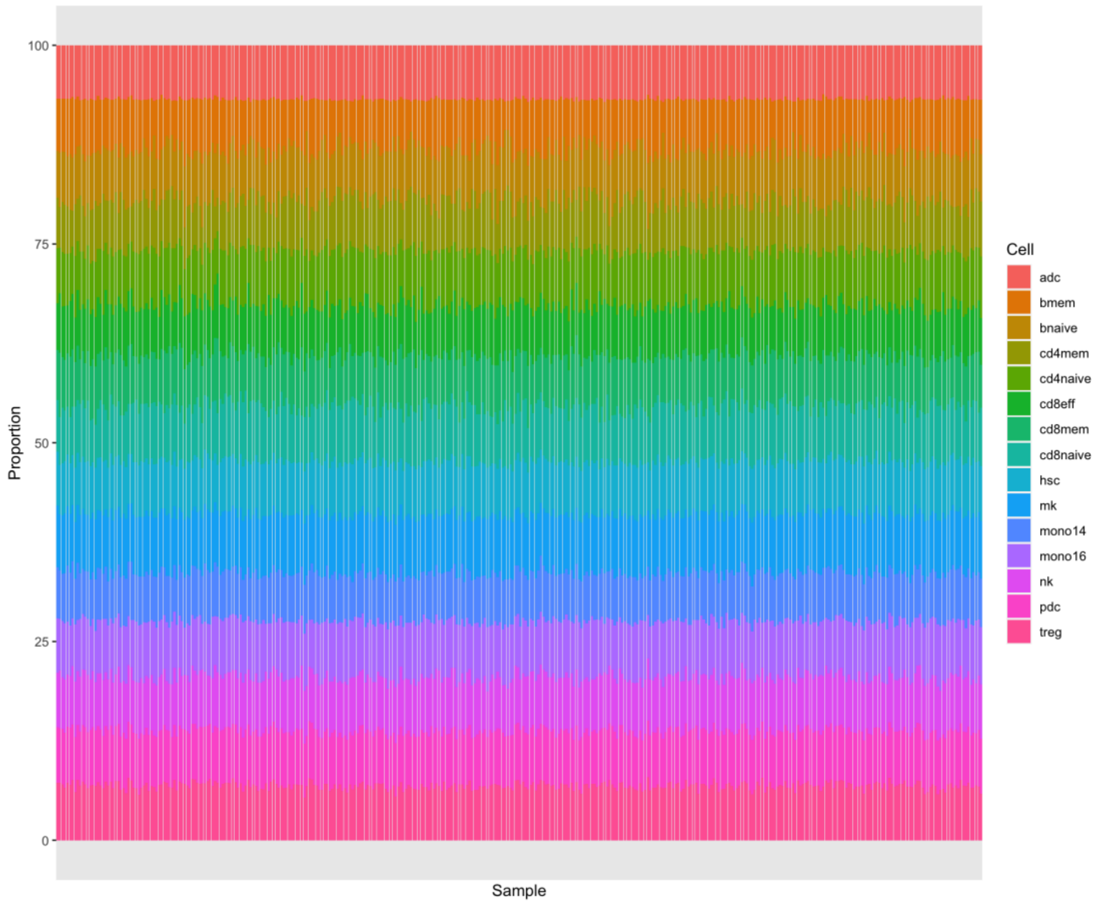


*Figure S10. The barplot showing HASCAD-predicted proportions of various cell types in 364 TCGA-LIHC bulk RNA-seq samples*

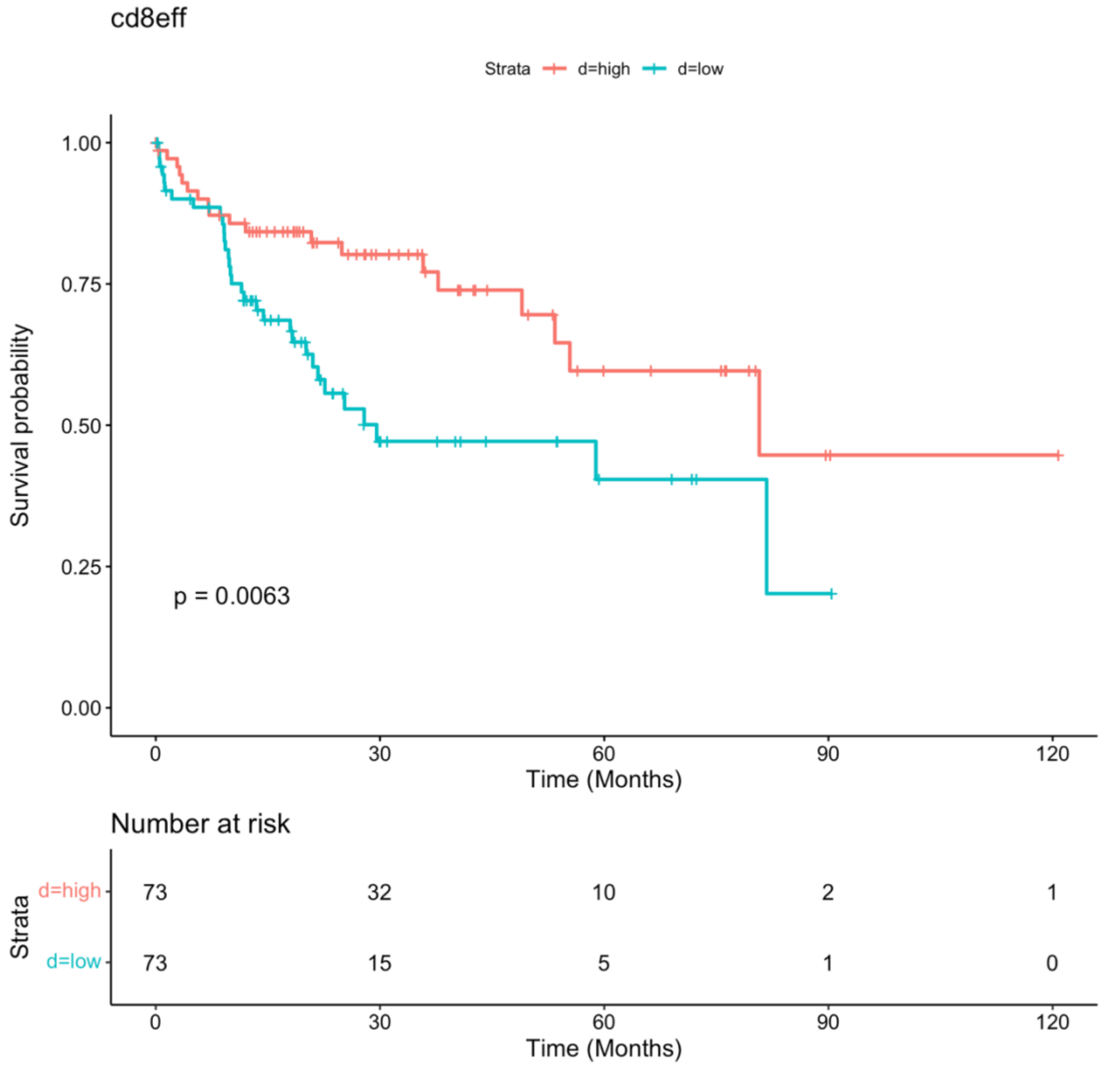


*Figure S11. A Kaplan–Meier plot showing the difference in overall survival of TCGA-LIHC patients between the high and the low groups of HASCAD-predicted proportions of effector CD8 T cells.*

*If the predicted proportions of effector CD8 T cells were larger than the upper quartile in all samples, these patients were assigned into the "high" group. On the other hand, if the predicted proportions of effector CD8 T cells were smaller than the lower quartile in all samples, these patients were assigned into the "low" group.*

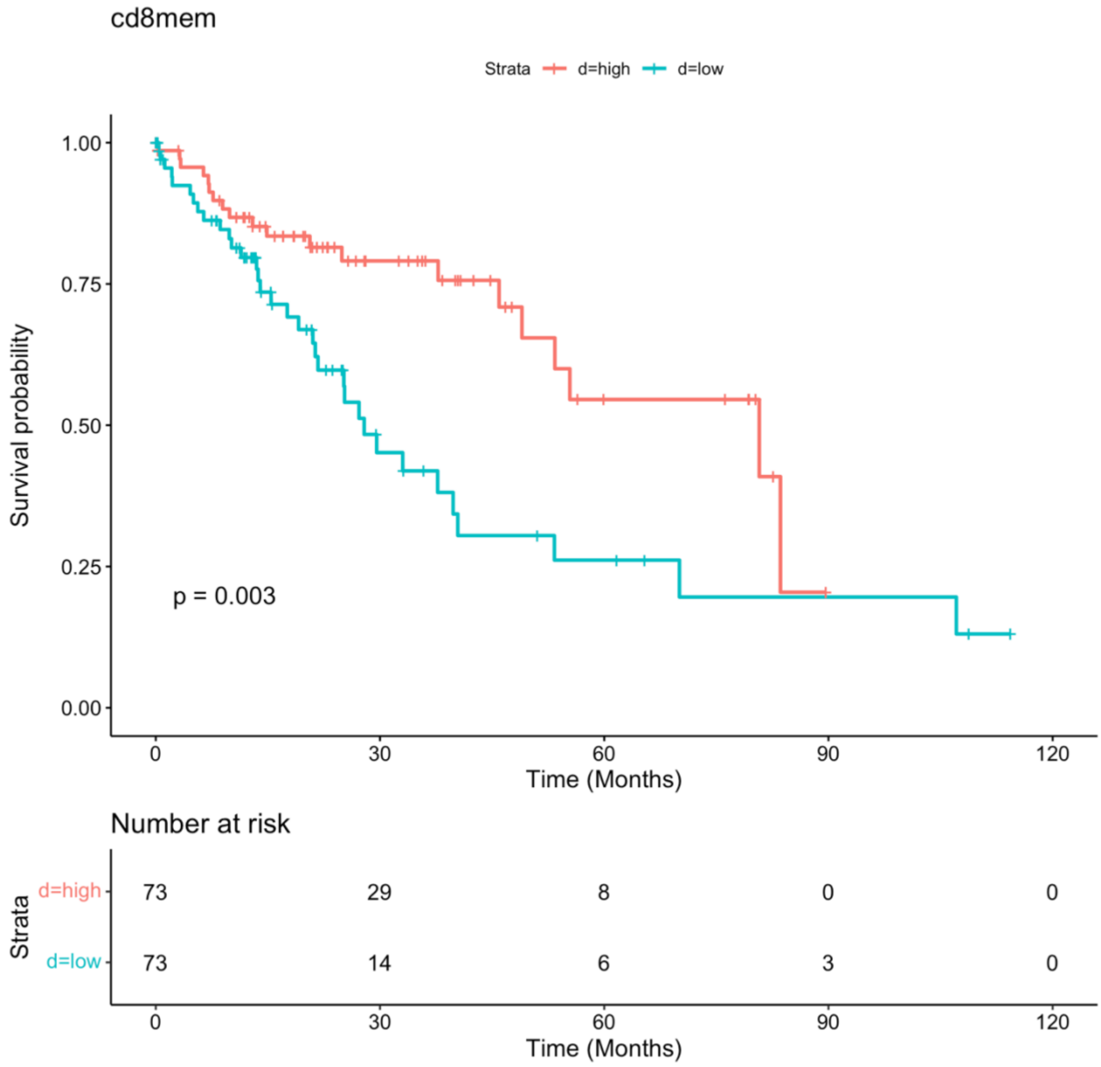


*Figure S12. A Kaplan–Meier plot showing the difference in overall survival of TCGA-LIHC patients between the high and the low groups of HASCAD-predicted proportions of memory CD8 T cells.*

*If the predicted proportions of memory CD8 T cells were larger than the upper quartile in all samples, these patients were assigned into the "high" group. On the other hand, if the predicted proportions of memory CD8 T cells were smaller than the lower quartile in all samples, these patients were assigned into the "low" group.*

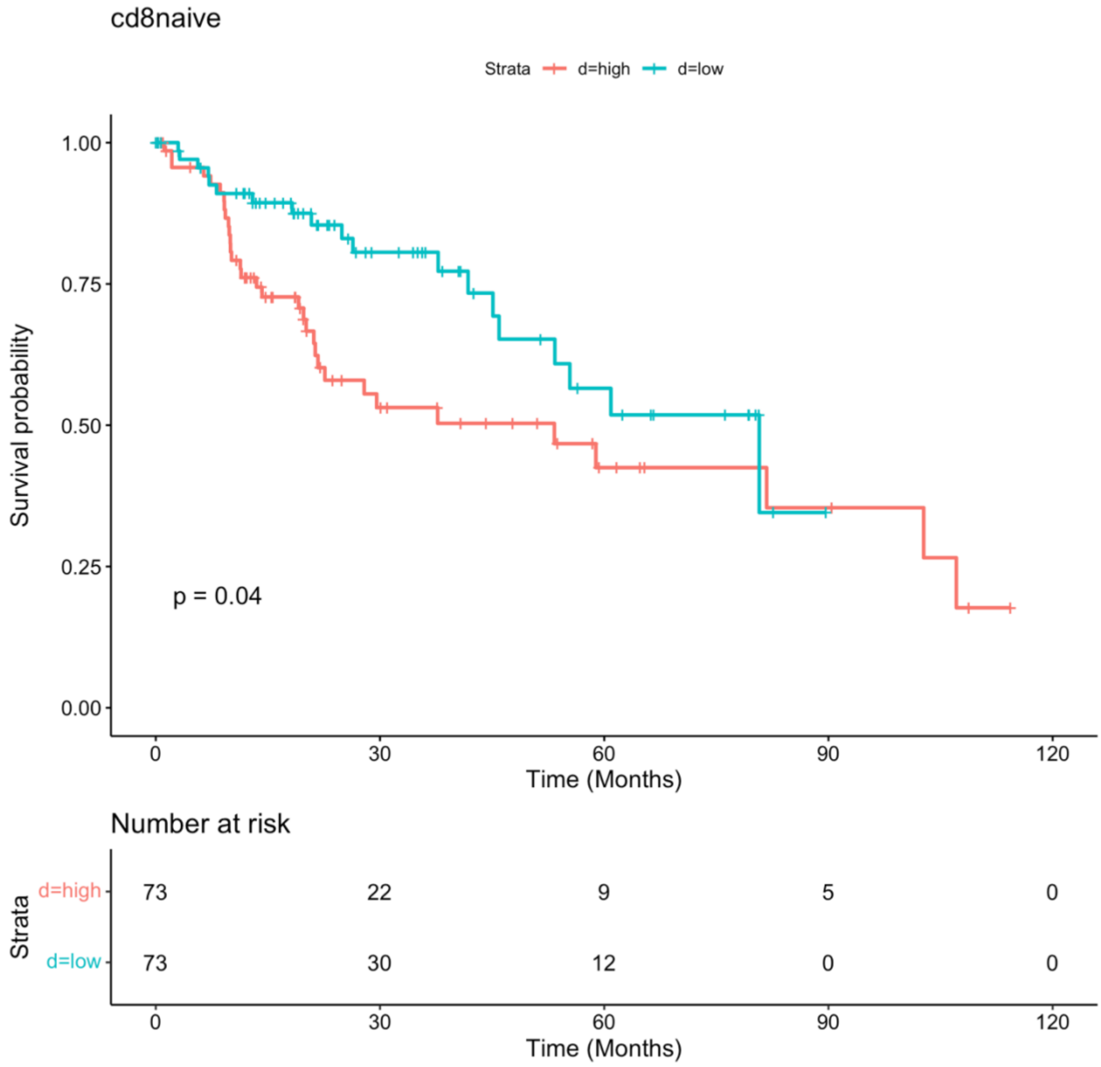


*Figure S13. A Kaplan–Meier plot showing the difference in overall survival of TCGA-LIHC patients between the high and the low groups of HASCAD-predicted naïve CD8 T cells.*

*If the predicted proportions of naïve CD8 T cells were larger than the upper quartile in all samples, these patients were assigned into the "high" group. On the other hand, if the predicted proportions of naïve CD8 T cells were smaller than the lower quartile in all samples, these patients were assigned into the "low" group.*

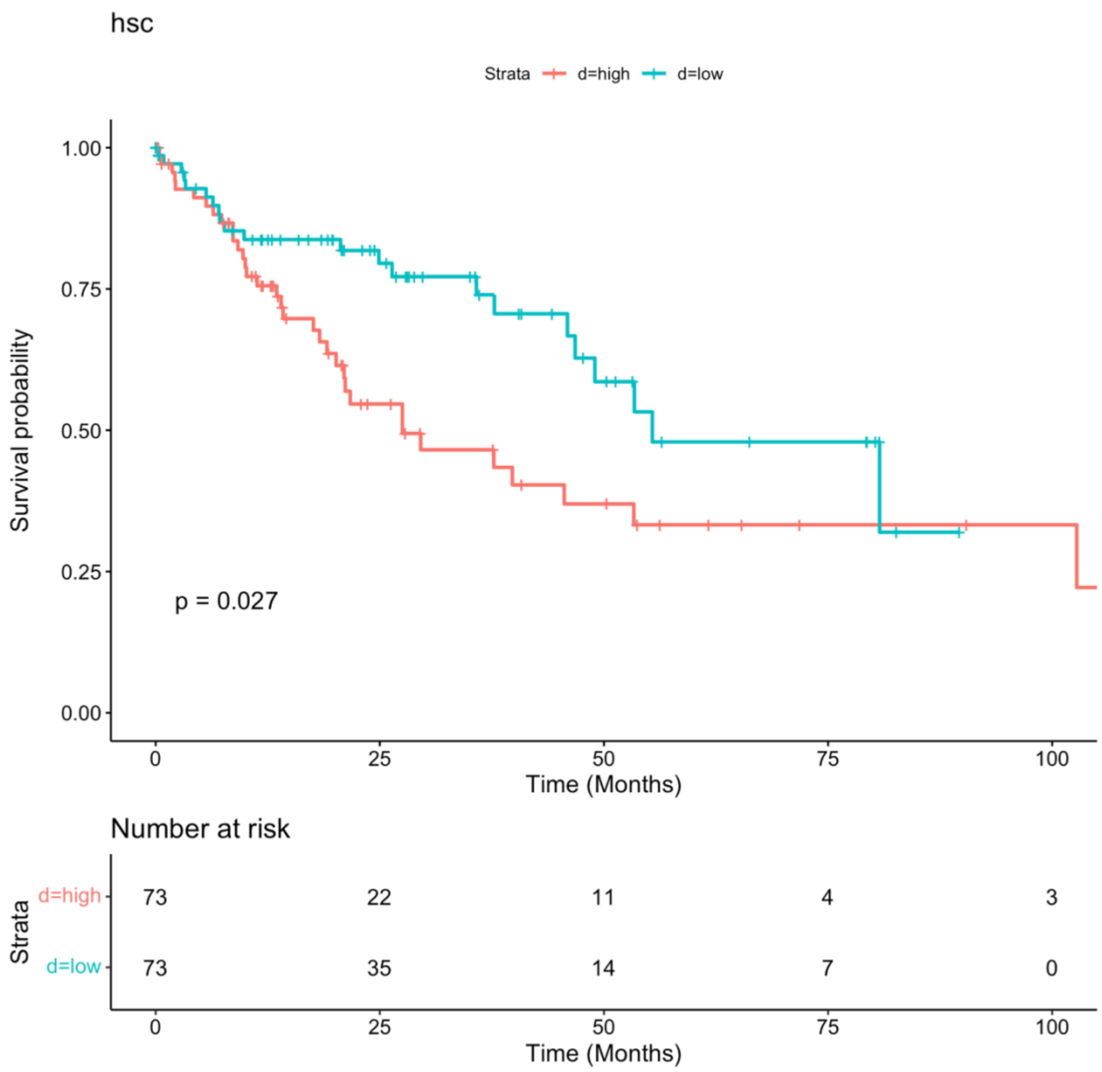


*Figure S14. A Kaplan–Meier plot showing the difference in overall survival of TCGA-LIHC patients between the high and the low groups of HASCAD-predicted proportions of hematopoietic stem cells.*

*If the predicted proportions of hematopoietic stem cells (HSCs) were larger than the upper quartile in all samples, these patients were assigned into the "high" group. On the other hand, if the predicted proportions of HSCs were smaller than the lower quartile in all samples, these patients were assigned into the "low" group.*
